# A New Antibody–Cytokine Construct Targeting Natural Killer Cells: An Immunotherapeutic Approach to Chronic Lymphocytic Leukemia

**DOI:** 10.3390/biom15010117

**Published:** 2025-01-13

**Authors:** Michela Flego, Mauro Andreotti, Francesca Romana Mauro, Maria Beatrice Arasi, Silvia Zamboni, Zuleika Michelini, Sara Pepe, Clementina Maria Galluzzo, Roberta Amici, Diego Moricoli, Chiara Mazzei, Alessandro Ascione, Alessandra Mallano

**Affiliations:** 1 National Center for Global Health, Italian Institute of Health, 00161 Rome, Italy; michela.flego@iss.it (M.F.); mauro.andreotti@iss.it (M.A.); zuleika.michelini@iss.it (Z.M.); clementina.galluzzo@iss.it (C.M.G.); roberta.amici@iss.it (R.A.); chiara.mazzei@studenti.unicz.it (C.M.); alessandro.ascione@iss.it (A.A.); 2 Hematology, Department of Translational and Precision Medicine, ‘Sapienza’ University, 00161 Rome, Italy; mauro@bce.uniroma1.it (F.R.M.); sara.pepe@uniroma1.it (S.P.); 3 Department of Oncology and Molecular Medicine, Italian Institute of Health, 00161 Rome, Italy; beatrice.arasi94@gmail.com; 4 Department of Neuroscience, Italian Institute of Health, 00161 Rome, Italy; silvia.zamboni@iss.it; 5 Diatheva s.r.l., Via Sant’Anna 131/135, 61030 Cartoceto, Italy; d.moricoli@diatheva.com

**Keywords:** chronic lymphocytic leukemia, natural killer cells, immunocytokine, IL15, antibody therapeutics, scFv

## Abstract

In chronic lymphocytic leukemia (CLL), natural killer (NK) cells show a dysfunctional phenotype that correlates with disease progression. Our aim was to restore NK cell functionality in CLL through a specifically targeted IL15-stimulating activity; IL15 targeting could, in fact, potentiate the activity of NK cells and reduce off-target effects. We designed and developed a cis-acting immunocytokine composed of an anti-CD56 single-chain Fragment variable (scFv) and IL15, labeled scFvB1IL15. scFvB1IL15 was tested in vitro on peripheral blood mononuclear cells (PBMCs) obtained from both different healthy donors (HDs) and CLL patients in order to evaluate its ability to target NK cells and enhance their activation and NK-mediated directed cytotoxicity. scFvB1IL15 specifically induced strong degranulation and cytokine and chemokine production in NK cells in both HD- and CLL patient-derived PBMC samples. Furthermore, compared to IL15 alone, it was able to induce higher levels of NKG2D- and NKp30-activating receptors and restore NK-mediated direct killing in the CLL patient-derived samples. The preliminary data presented in this work suggest that IL15’s targeting of NK cells via scFvB1 potentiates the effects of IL15 and that scFvB1IL15 can be a useful agent for overcoming NK functional gaps and contribute to NK-cell-based immunotherapies.

## 1. Introduction

CLL is characterized by chronic clonal expansion of mature CD19-expressing B-lymphocytes. Recent novel targeted therapies have greatly expanded the treatment choices for many patients, such as Bruton tyrosine kinase and BCL-2 inhibitors (i.e., ibrutinib, acalabrutinib, zanubrutinib, pirtobrutinib, and venetoclax); these therapies have demonstrated potent antitumor activity with prolonged remission. However, CLL remains an incurable disease.

In this context, novel therapeutic approaches including those that potentiate the killing of tumor cells by the immune system’s cell action are needed to improve the eradication of malignant cells. The benefit of CAR-T cells has been observed in some relapsed/refractory patients with CLL [1]. Recent studies have investigated the efficacy of autologous or allogenic NK-cell-based therapy [2,3,4].

In patients with progressive CLL, NK cell dysfunction is often observed, although little is known about the mechanisms leading to this dysfunction. Reduced effector responses such as direct cytotoxicity [5,6] have been described, including reduced production of pro-inflammatory cytokines such as IFNγ [7]. Various studies have identified some factors that can contribute to NK cell deficiencies. A dysregulated expression of activating/inhibitory surface receptors [8,9]—in particular, a decrease in the NKG2D-activating expression—has been reported [5,6].

Interestingly, a reduction in fully mature and cytotoxic NK cells [10], as well as higher percentages of more immunomodulant CD56bright NK cells in patients with CLL with a higher beta 2-microglobulin level [11], has been observed.

Several mechanisms underlying NK dysfunction are linked to the tumor microenvironment. CLL cells can produce suppressing soluble factors, such as TGF-β1, and express a series of inhibitory ligands that can directly impair NK cells [6,12,13,14]. Moreover, CLL patients show impaired regulatory T cells (Tregs) and myeloid-derived suppressor cells (MDSCs), which suppress NK cells. In addition, novel agents may exert negative effects on the cytotoxicity, proliferation, and survival of NK cells [14,15].

Several cytokines, such as IL2, IL15, IL12 IL18, IL21, and their combinations, have been found to effectively stimulate in vitro NK cells in CLL patients and reverse their impairment [12,13,16]. Interestingly, IL15 is not associated with the promotion of immunosuppressive regulatory T cells, in contrast to other NK-activating cytokines such as IL2, IL18, and IL21.

However, the administration of IL15 in clinical settings shows some limitations, including its short half-life and severe adverse events due to the high doses required to reach functional responses. In recent years, new formulations of IL15 and IL15-based constructs have been developed with increased half-life, strength, and potencies of biological activities in order to minimize the risk of toxicity and maximize therapeutic potential. Modifications made to the IL15 molecule include (but are not limited to) empowering mutations; conjugation to receptor α (IL15Rα, sushi domain) or other cytokines (to potentiate activity); linking to the PEG or IgG FC domain (to increase half-life); and conjugation with antibodies targeting the receptors on effectors or target cells [17,18,19].

IL15 and some of its derivatives (i.e., NIZ985; ALT-803; Receptor-Linker-IL15, RLI) has been investigated in different clinical trials, with preliminary data suggesting its role as a single agent, or in cellular treatment approaches for solid and hematological malignancies, including CLL [17,18,19].

A controversial issue regarding the use of IL15-based therapy for treating lymphoproliferative disorders is the pathogenic role of IL15 in these malignancies [20]. IL15 can promote B-cell proliferation and lymphomagenesis, both as a secreted cytokine or a cytokine trans-presented by the surrounding cells in the germinal centers [21,22].

The objective of our work is to demonstrate that, thanks to conjugation to an antibody specific for NK cells subpopulation, the action of IL15 can be specifically targeted, reducing unwanted side effects due to the action of the cytokine on off-target cellular subpopulation or reducing the doses needed to obtain a desired effect on a specific population.

For this purpose, IL15 was conjugated with a recombinant human anti-CD56 antibody through a flexible linker, thus allowing simultaneous binding to CD56 and IL15 receptors on the same cell. Firstly, we tested this bi-functional construct on PBMCs derived from healthy donors and subsequently on PBMCs derived from patients affected by progressive CLL, as an example of a disease that induces phenotypic and functional alterations of NK cells.

We found that this construct was able to direct IL15 activity to the NK subpopulation, potentiate NK cell functionality, and restore NK-mediated cytotoxicity in CLL PBMCs, suggesting its potential application as an agent for sustaining the anti-tumoral activity of NK cells in CLL and promoting NK-cell-based immunotherapies.

## 2. Materials and Methods

### 2.1. scFvB1, CD56 Antigen, and Cell Lines

The scFvB1 antibody specific for the CD56 antigen was isolated from our IORISS naive human antibody library; its characterization has been previously described [23,24].

The CD56 antigen used for direct ELISA with the immunocytokine was a purified recombinant protein corresponding to the extracellular domain of human CD56 with 6xHis-tag (CD56ecd); it was produced in *E. coli* by Biologics International Corp, a protein manufacturer (Indianapolis, IN, USA).

Glucose oxidase (GO) antigen was purchased from Sigma-Aldrich (St. Louis, MO, USA).

NK92 is an interleukin-2 (IL-2)-dependent natural killer cell line and was a kind gift of Professor L. Moretta, who obtained the cells from the ATCC repository (ATCC; CRL-2407).

K562 is a human immortalized myelogenous leukemia cell line, which was obtained from the ATCC repository (CCL-243).

The cells were grown under standard conditions for mammalian cell cultures. The basic medium for cell culturing consisted of RPMI-1640 (EuroClone, Milan, Italy) supplemented with 10% heat-inactivated fetal bovine serum (FBS), L-glutamine (2 mM), and penicillin (100 U/mL)/streptomycin (100 U/mL). All of these components were purchased from Euroclone (Milan, Italy). The recombinant human IL-2 for NK-92 culture (50 ng/mL) and IL15 (10 ng/mL) used in some experimental procedures were purchased from Cell Guidance Systems (CellGS, Cambridge, UK).

### 2.2. PBMC Isolation from HD and Patient Samples

The PBMCs were purified from buffy coats obtained from the Transfusion Center of the “Sapienza” University of Rome, as waste material derived from the procedures of plasma/platelet/red blood isolation from the whole blood of HDs, who provided written informed consent. According to the Italian law (Legislative Decree of the Italian Ministry of Health of 25 January 2001, Gazzetta Ufficiale, 3 April 2001), the donor ages ranged between 21 and 60 years for women and 21 and 65 years for men. All donors were healthy, non-medicated, consenting adults who were prescreened for exposure to infectious agents.

The CLL patient blood samples were obtained from the Institute of Hematology in the Department of Translational and Precision Medicine at the University of Rome, “Sapienza” Italy, after written consent was obtained from the patients. The use of PBMCs from patients was approved by the Ethical Committee (EC) of the Istituto Superiore di Sanità and the EC of the University of Rome “Sapienza”/Azienda Policlinico Umberto I of Rome, Italy.

The patients with CLL were previously untreated and showed progressive disease.

PBMCs were isolated from the heparinized blood samples of the HDs or CLL samples via Ficoll–Hypaque density gradient centrifugation and cultured as previously described for mammalian cells.

### 2.3. Molecular Modeling

The 3D structural simulation of scFvB1IL15 and the extracellular domain of both human CD56 and IL15R structures were built with the Phyre2 Protein Fold Recognition Server [25], and the homology model structure was retrieved for analysis. Three-dimensional models of scFvB1IL15 and the extracellular domain of CD56 or IL15R were submitted to the ClusPro protein–protein docking server [26] to observe the most probable interaction between the two inferred 3D structures and visualized using the Molegro Molecular Viewer software for the coherent contact interface.

### 2.4. Genetic Construction, Expression, and Purification of Immunocytokine scFvB1IL15

In order to isolate IL15 cDNA, the PBMCs isolated from the HDs were placed in 24-well plates at 2 × 10^6^ cells/mL in the presence of IL2 (50 ng/mL) in RPMI 20% FBS overnight (ON). On the next day, the supernatant was harvested, and adherent macrophages were activated with 100 ng/mL of LPS (Sigma, St. Louis, MO, USA) in RPMI for 24 h. Then, the macrophages were harvested and used for mRNA extraction using a QuickPrep Micro mRNA Purification Kit (Sigma-Aldrich, St. Louis, MO, USA) according to the manufacturer’s instructions. The extracted mRNA (1 μg) was reverse-transcribed for double-strand cDNA synthesis using a SmarTer PCR Synthesis kit (Clontech, Takara Bio, Saint-Germain-en-Laye, France). Then, DNA coding for IL15 was amplified by PCR from the total cDNA, while the scFvB1 coding sequence was amplified by PCR from the IORISS phagemide vector [24] with tailored primers.

The complete immunocytokine gene (1250 bp) was synthetized using DNA recombinant and ligation techniques. The final configuration includes the 6xHis tag, scFvB1 sequence, a 25 aa (GGGGS)_5_ linker, the IL15 sequence, and the Flag tag. The gene was cloned in pcDNA3.1 vector at the EcoRV/XhoI restriction sites for protein expression in a eukaryotic system already modified with an IL-2 signal peptide sequence for protein secretion in the culture medium, which was kindly provided by Prof. Ferrini (IRCCS A.O.U. San Martino—IST, Istituto Nazionale per la Ricerca sul Cancro, Genova), and cloned at the HindIII/EcoRVrestriction sites.

The construct was transplanted into competent *E. coli* cells, and the resulting amplified and purified plasmid was used for transfection of 293LentiX cells for the transient production of the fusion protein. In brief, the cells (3.5 × 10^6^) were transfected with 10 µg of plasmid DNA per 10 cm of the tissue culture plate using the jetPRIME^®^ DNA Transfection Reagent (Polyplus-transfection, S.A., Illkirch, France) according to the manufacturer’s instructions. The supernatants were collected 72 h following the transfection to evaluate antibody production and binding activity using ELISA assays. His-tagged protein was purified via immobilized metal affinity chromatography using Ni2+-nitriloacetic acid agarose (Qiagen, Hilden, Germany). The ScFvB1IL15 purified protein was eluted with 250 mM imidazole in PBS 1×, dialyzed against PBS 1×, and stored at −80 °C.

### 2.5. Western Blotting

A total of 15 µL of the purified scFvB1IL15 was separated using 12% SDS-PAGE and then transferred to a nitrocellulose membrane. After blocking with 2% non-fat milk in PBS (MPBS), the membrane was washed and incubated for 1 h at R.T. with the monoclonal mouse anti-hIL15 antibody (cod. 500-M15, PeproTech, London, UK) in 2% MPBS; after three washings with PBS Tween 0,1%, the membrane was incubated with the secondary HRP-conjugated anti-mouse antibody (1:1000 dilution, cod. P0447, Dako, Glostrup, Denmark) in 2% MPBS. The filter was developed using the DAB (3,3′-diaminobenzidine) Color Development Solution (BioRad, Hercules, CA, USA).

### 2.6. ELISA Using Recombinant CD56 Protein

ELISA MaxiSorp Nunc 96-well immunoplates (Thermo Fisher Scientific, Rome, Italy) were coated ON with 50 µL/well of 10 µg/mL CD56ecd or GO irrelevant antigen in PBS at 4 °C. On the next day, a blocking solution consisting of 2% MPBS was added, and after 2 h, the plates were washed three times with PBS. The plates were incubated for 2 h at room temperature (RT) with 50 µL of the supernatants containing soluble scFvB1IL15 immunocytokine, anti-Flag M2 antibody (2.5 µg/mL, cod. F3165, Sigma, St. Louis, USA,), and HRP-conjugated anti-mouse antibody (5 μg/mL, cod. P0447, Dako, Denmark). All antibodies were re-suspended in 2% MPBS.

The reaction was visualized with 50 μL/well of 3.3′-5.5′-tetramethylbenzidine (TMB) substrate (Surmodics, Eden Prairie, MN, USA) and stopped by adding 50 µL/well of a liquid stop solution (Surmodics, USA). The reaction was detected using an ELISA reader (Model 680 microplate reader, Biorad, Hercules, CA, USA). The results are expressed as A = A (450 nm)−A (620 nm).

### 2.7. Determination of Binding Affinity

The determination of antibody affinity was performed via equilibrium saturation analysis using a competitive ELISA assay according to S. Rath et al. [27], with minor modifications.

A first ELISA step was performed to determine the antibody concentration at which half of the target was present in the bound state; that is, the half maximal effective concentration (EC50). Microtiter plates (Nunc, Roskilde, Denmark) were coated with 50 μL of the CD56ecd antigen (10 μg/mL) in a PBS buffer and incubated ON at 4 °C. The microtiter plates were washed three times with 0.05% Tween PBS (TPBS) and blocked with MPBS for 2 h at RT. The microtiter plates were then washed three times with TPBS and incubated for 2 h at RT with 50 µL of the serial dilutions of scFvB1IL15 immunocytokine in MPBS, together with 10 μL of a freshly prepared mixture consisting of an anti-FLAG M2 (12.5 μg/mL, cod. F3165, Sigma-Aldrich, St. Louis, USA) and an anti-mouse HRP-conjugated antibody (22 μg/mL, cod. P0447, Dako, Germany). After the incubation period, the plates were washed three times with TPBS and three times with PBS. The color development system was added and the color intensities were measured as indicated above. The EC50 was calculated based on obtained absorbance values.

A second ELISA step was performed to determine the ratio of the concentration of free antibody at equilibrium. The wells were coated with 0.5 μg of CD56ecd protein. The day after, the serial dilutions of the same antigen were incubated in a solution with scFvB1IL15 at the determined EC50 dilution point (calculated from the first ELISA step) for 1 h to reach equilibrium.

After the blocking step, the solution with the antigen at varying concentrations and the immunocytokine was added to the designated antigen-coated wells, together with a freshly prepared mixture consisting of an anti-FLAG M2 (12.5 μg/mL, cod. F3165, Sigma-Aldrich, USA) and an anti-mouse HR-conjugated antibody (22 μg/mL, cod. P0447, Dako, Germany). The microtiter plates were rinsed thoroughly, the substrate solution applied, and then the enzyme reaction was developed and measured as described above. The unbound immunocytokine was determined based on the ELISA values; the molar inhibitor concentration required for 50% inhibition (IC50) represents an estimate of the average antibody affinity. Analysis of the results was performed using the GraphPrism program.

### 2.8. ELISA Sandwich for Determination of Purified Protein Concentration

We quantified the scFvB1IL15 purified protein using a sandwich ELISA, with the commercial recombinant human IL15 cytokine from Cell Guidance Systems as a standard, which has an expected specific activity of 2 × 10^5^ units/mg. Since this cytokine was used as the positive control in all functional experiments with PBMCs from the HDs and CLL patients, this quantification method allowed for an accurate comparison of the activity of scFvB1IL15 and free IL15.

The monoclonal mouse anti-hIL15 antibody (cod. 500-M15, PeproTech, London, UK) was diluted in PBS at 10 μg/mL, added to a 96-well plate (Nunc, Roskilde, Denmark), and incubated ON as a coating. Then, the recombinant human IL15 was used as a standard ranging from 1000 to 0.5 ng/mL. After several washes, proteins were detected with 1 μg/mL of biotinylated rabbit anti-hIL15 (500-P15bt, PeproTech, London, UK) and then with HRP-conjugated streptavidin (Dako, Denmark) diluted 1:1000 in 2% MPBS. The reaction was visualized as previously described.

The obtained OD versus commercial IL15 concentrations or scFvB1IL15 dilutions were graphed, and logarithmic curves were extrapolated using GraphPad Prism 10.2.2. We chose the point at 0.3 OD in the linear portion of both curves and calculated the corresponding concentrations of IL15 and scFvB1IL15 dilution according to the equation of the extrapolated curves. Finally, the scFvB1IL15 concentration was calculated as the concentration of IL15 at the 0.3 OD/the scFvB1IL15 dilution at the same OD (Supplementary Figure S1).

### 2.9. Construct Binding to Native CD56

To evaluate the ability of scFvB1IL15 to still recognize CD56 on the cell surface, we performed flow cytometry binding experiments on NK92 cells.

First, cells in the exponential phase of growth were collected and then carefully washed in PBS and pelleted. About 2.5 × 10^5^ cells were re-suspended in PBS containing different concentrations of scFvB1IL15 and incubated for 1 h at RT. After washing, the cells were re-suspended in PBS containing M15 mouse anti-hIL15 (cod. 500-M15, Peptotech, London, UK) diluted 1:1000 in PBS 1× for 30 min at 4 °C. After washing, the cells were incubated with 6 μg/mL of an FITC-goat anti-mouse IgG (cod. 31569, ThermoFischer scientific, Rome, Italy) for 30 min at 4 °C. As a positive control, cells were marked with commercial anti-CD56 FITC-conjugated antibody (Clone NCAM16.2, cod. 340410, Becton Dickinson BD, Franklin Lakes, NJ, USA) for 30 min at 4 °C. After staining, the cell samples were washed, maintained at 4 °C, and immediately analyzed. Fluorescence compensation was determined using the samples stained with anti-glucose oxidase scFv and goat FITC-conjugated anti-mouse secondary antibody.

To confirm that the binding we observed with scFvB1IL15 was specific and attributable to the antibody portion, we also performed a competition experiment where NK92 cells were pre-incubated with scFvB1 at 250 μg/mL or an irrelevant anti-glucose oxidase scFv (scFvGO) [28] together with scFvB1IL15 at 1 μg/mL. Finally, the cells were marked with anti-hIL15 and FITC-conjugated anti-mouse secondary antibodies, as described before.

To verify that scFvB1IL15 was able to recognize NK cells in a mixed population under physiological conditions, PBMCs at 1 × 10^6^ cells/sample were collected, washed with PBS, and marked with the LIVE/DEAD™ Fixable Aqua Dead Cell Stain reagent (Invitrogen, Waltham, MA, USA) in order to exclude dead cells during the FACS analysis. The cells were then washed, re-suspended in PBS containing 1 μg/mL of scFvB1IL15, and incubated for 1 h at 4 °C to avoid IL15R-mediated internalization of the construct [29]. After washing, the cells were re-suspended in PBS containing anti-His (MCA1396, BioRad, CA, USA) diluted 1:1000 in PBS 1× for 30 min at 4 °C. After washing, the cells were incubated with 6 μg/mL of an FITC-goat anti-mouse IgG (ThermoFischer scientific, 31569) for 30 min at 4 °C. As a positive control, PBMCs were marked with commercial anti-CD16-PE (Clone B73.1, cod. 561313) and anti-CD56-FITC (Clone NCAM16.2, cod. 340410) (Becton Dickinson BD, NJ, USA) for 30 min at 4 °C. After staining, the cell samples were washed, maintained at 4 °C, and immediately analyzed.

### 2.10. CTLL-2 Growth Curve

The determination of cytokine functionality was performed based on the growth curve of the CTLL-2 cell line in 96-well plates and by determining the relative EC50. In particular, the cells were seeded at a density of 10^4^ cells per well in the 96-well plates in 200 of RMPI 10% FBS, cultured in the presence of different concentrations of scFvB1IL15 or the commercial IL15 (CellGS, Cambridge, UK) ranging from 50 to 0 ng/mL, and incubated at 37 °C with 5% CO_2_. After 48 h, Premix WST-1 (Takara) (20 μL/well) was added, and the plates were incubated for 90 min at 37 °C with 5% CO_2_. Absorbance at 450 nm was measured using a Model 680 microplate reader, Biorad (CA, USA). Data analysis and EC50 calculation were carried out using the GraphPad Prism 10.2.2 program.

### 2.11. Intracellular Cytokine Staining

PBMCs were isolated from the HDs’ buffy-coat samples and the CLL patients’ blood samples via Lympholyte-H (Cedarlane, Burlington, ON, Canada) density centrifugation and incubated ON (37 °C, 5% CO_2_) at a concentration of 2 million/mL in RPMI 10% FBS without any treatment (null) or in the presence of IL15 or scFvB1IL15 at 10 ng/mL. K562 cells were marked with CFSE (Invitrogen, Waltham, USA) according to the manufacturer’s instruction and incubated ON in RPMI 10% FBS. On the next day, the PBMCs were plated at 250,000 cells/150 µL/well in 96-well plates with RPMI 10% FBS in the presence or absence of commercial IL15 or immunocytokines at 10 ng/mL (4 replicates per condition), together with CD107aPerCP-Cy5.5 (Clone H4A3, cod. 328616, Biolegend, San Diego, CA, USA). The plates were incubated for 1 h; then, 50 µL of K562 cells were added at 10,000 cells/patient and the plates were further incubated at 37 °C for 1 h. GolgiStop (1:1500, Becton Dickinson BD, Franklin Lakes, NJ, USA) and GolgiPlug (1:1000, BD Biosciences) were added, and the plates were further incubated for 4 h. After incubation, the cells were harvested, washed, and treated with the LIVE/DEAD™ Fixable Aqua Dead Cell Stain reagent (Invitrogen) in order to exclude dead cells during the FACS analysis. Then, the cells were stained for surface antigens with CD19PE (Clone HIB19, cod. 555413, BD, Franklin Lakes, NJ, USA), CD14PE (Clone M5E2, cod. 555398, BD, Franklin Lakes, NJ, USA), and CD3APC-Cy7 (Clone SK7, cod. 557832, BD, Franklin Lakes, NJ, USA) and permeabilized and stained for intracellular antigens with MIP1b-PeCy7 (Clone D21-1351, cod. 560687, BD, Franklin Lakes, NJ, USA), IFNγBV421 (Clone B27, cod. 562988, BD, Franklin Lakes, NJ, USA), and TNFαPECF594 (Clone MAb11, cod. 562784, BD, Franklin Lakes, NJ, USA). The NK population was identified excluding CD19/CD14/CD3+ cells, as direct staining with CD56 antibody was not possible due to the partial or total loss of binding after the scFvB1IL15 treatment.

### 2.12. NK-Specific Surface Receptor Staining

PBMCs from the HD and CLL patient samples were isolated and incubated ON (37 °C, 5% CO_2_) at a concentration of 2 million/mL in RPMI 10% FBS without any treatment (null) or with supplementation with IL15 (CellGS, Cambridge, UK) or scFvB1IL15 at 10 ng/mL. K562 cells were marked with CFSE (Invitrogen) according to the manufacturer’s instruction and incubated ON in RPMI 10% FBS. On the next day, the PBMCs were plated at 250,000 cells/150 µL/patient in 96-well plates with RPMI 10% FBS in the presence or absence of commercial IL15 or immunocytokines at 10 ng/mL (4 replicates per condition); then, 50 µL of the K562 cells were added at 10,000 cells/patient and the plates were incubated at 37 °C for 1 h. After incubation, the cells were harvested, washed, and treated with the LIVE/DEAD™ Fixable Aqua Dead Cell Stain reagent (Invitrogen) in order to exclude dead cells during the FACS analysis. Then, the cells were stained for surface antigens with CD19/CD14PE, CD3APC-Cy7, and NKp30 BV421 (Clone p30-15, cod. 563385, BD, Franklin Lakes, NJ, USA); NKG2D APC (Clone 1D11, cod. 558071, BD, Franklin Lakes, NJ, USA); and NKp46 PeCy7 (Clone 9E2, cod. 562101, BD, Franklin Lakes, NJ, USA). The NK population was identified excluding CD19/CD14/CD3+ cells, since direct staining with CD56 antibody was not possible because of the partial or total loss of binding after the scFvB1IL15 treatment.

### 2.13. Direct NK Cytotoxicity Assay

K562 cells were marked with Cell Trace Violet (CTV, Invitrogen) according to the manufacturer’s instruction and incubated ON in RPMI 10% FBS. PBMCs from the HD and CLL patient samples were isolated and incubated ON (37 °C, 5% CO_2_) in RPMI 10% FBS without any treatment (null) or with supplementation with IL15 or scFvB1IL15 at 10 ng/mL. On the next day, the PBMCs were co-cultured with the K562 cells at 50:1 (250,000 PBMCs/well with 5000 K562 cells/well), 25:1, 12.5:1, 6.25:1, and 3.12:1 ratios in the presence or absence of IL15 or immunocytokines at 10 ng/mL in 96-well plates (4 replicates for each condition) and incubated for 5 h.

After incubation, the cells were harvested, washed, and then re-suspended in PBS with 1 μg/mL of 7-aminoactinomycin D (7AAD, Thermo Fisher Scientific, Rome, Italy).

The gating strategy was performed as previously described [30]. The specific lysis of target cells measured at different effector versus target ratios allows for the calculation of lytic units as LU_30_/10^7^ cells. The percentage of specific lysis was plotted as a function of the actual E:T ratio. A linear regression was calculated from the plot, and the equation of the trendline was used to calculate the E:T ratio required to lyse 30% of K562 cells. The lytic activity is defined as the number of lytic units contained in 10^7^ effector cells: LU_30_/10^7^ = 10^7^/T × Xp, where T is the number of target cells; p is the reference lysis level (30%); and Xp is the E:T ratio required to lyse 30% of the targets [31].

### 2.14. Flow Cytometer Acquisition and Analysis

The samples from the binding experiments using NK92 cells were acquired with a FACScan (BD, Franklin Lakes, NJ, USA) equipped with a 15 mW, 488 nm argon laser.

The samples from the ex vivo experiments using the PBMCs from the HDs and CLL patients were analyzed using a Gallios flow cytometry system (Beckman Coulter, Brea, CA, United States) with multi-lasers (emitting light at 405, 488, and 640 nm) and multiple detectors. The data were processed with the Kaluza analysis software 2.1 (Beckman Coulter, Brea, CA, USA).

### 2.15. Statistical Analysis

Statistical analysis of the experimental results was performed using SPSS Statistics version 28. As the collected data were generally not normally distributed, we report the medians and ranges for the parameter values. Non-parametric statistical methods were used to analyze the data.

The Wilcoxon test was used for comparison between the experimental and control conditions; the Mann–Whitney test was used for comparison between the CLL patients and HDs; correlations were analyzed using Spearman’s rank correlations. Significance was assessed at the *p* = 0.05 level.

## 3. Results

### 3.1. Design, Synthesis, and Purification of the scFvB1IL15 Immunocytokine

**Figure 1:**
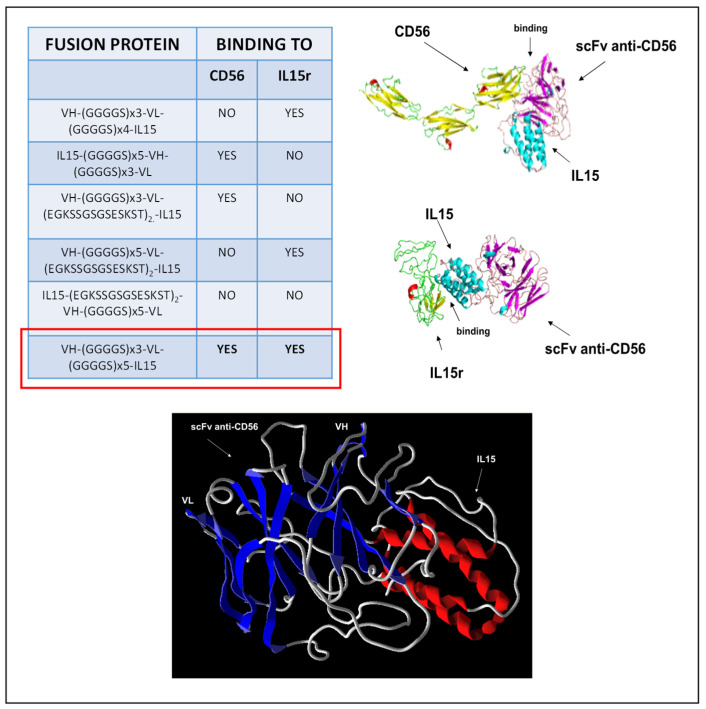
Bioinformatic analysis for scFvB1IL15 (fusion protein) construction. On the left, the results of the bioinformatic analysis of the most probable interaction and coherent contact interface between the inferred 3D structures of scFvB1IL15 (different constructs) and IL15R or CD56. On the right, the three-dimensional models of interaction with the ligands are shown. The docking simulation shows that the final construct has enough flexibility to allow both IL15 and scFvB1 binding to the IL15 receptor and CD56 antigen, respectively. The putative three-dimensional structure of the chosen construct is shown at the bottom and shows the two immunoglobulins domains which correspond to the VH and VL fragments.

We performed a bioinformatic analysis of different constructs with different linker compositions or spatial orientations to design the scFv anti-CD56/IL15 immunocytokine. This was performed such that the final construct had enough flexibility to allow IL15 and scFvB1 binding to the IL15 receptor and CD56 antigen, respectively. First, the hypothetical three-dimensional structures were built, and then we performed a docking analysis to verify the interactions between IL15 and its receptor and between scFvB1 and the CD56 antigen. The interactions were compared to those observed with the unconjugated IL15 and scFvB1 with their respective ligands to verify their accuracy. The only construct that seemed to have both coherent contact interface for scFvB1 and IL15 was constituted by scFvB1 at the N-terminal and conjugated to IL15 by a 25 aa peptide linker with the (GGGS)_5_ composition. This linker length likely lends the immunocytokine its flexibility to allow both IL15 and scFvB1 to reach the right steric conformation and bind to their respective receptors (Figure 1).

**Figure 2:**
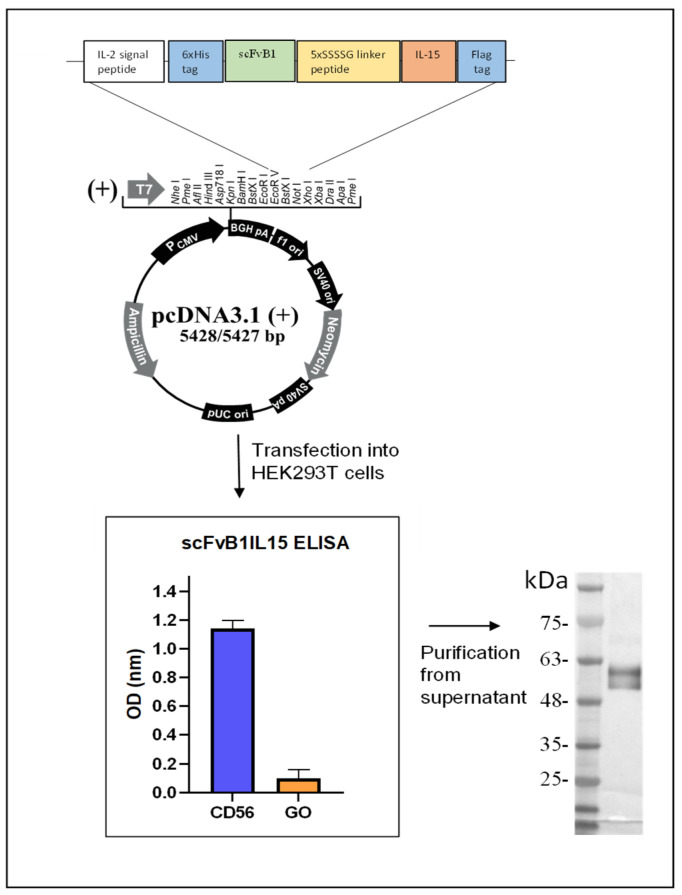
ScFvB1IL15 molecular characterization. The upper part of the figure shows a schematic representation of the scFvB1IL15 gene with 6His and Flag tags, cloned into the eukaryotic vector pcDNA3.1. Below, on the left, the OD results obtained from the ELISA performed on CD56ecd (blue) and GO (orange) antigens with the supernatants from the HEK293T cells transiently transfected with scFvB1IL15 cloned in the pcDNA3.1 vector are shown. On the right, the WB analysis of the purified scFv1B1IL15 protein is shown. The molecular weights of the BlueElf Prestained Protein Marker (JenaBioscience, Jena, Germany) are reported.

The gene encoding for the constructs (1250 bp) was cloned in a pcDNA3.1 eukaryotic vector for protein expression in 293LentiX cells. The supernatant from the transient transfections was tested using ELISA against the CD56 antigen, with glucose oxidase as the irrelevant antigen, resulting in good protein production. The protein was purified from the supernatant via affinity chromatography and analyzed using Western blot (Figure 2). The purified protein showed the expected weight of 50 kDa; the two bands correspond to two different glycosylation isoforms [32]. This protein was quantified using a sandwich ELISA, as described in the Methods section in relation to the recombinant human IL15 for functional assays (Supplementary Figure S1).

### 3.2. Evaluation of scFvB1IL5 Molecular Properties

After we obtained the purified scFvB1IL15 protein, we investigated whether the construct retained the ability to specifically bind to the CD56 antigen (antibody arm) and induce IL15-mediated biological effects (cytokine arm).

First, we measured its binding affinity to the CD56ecd antigen using a two-step ELISA, as described in the Methods section. The calculated dissociation constant (Kd) was 1.86 × 10^−8^ moles/liter (M) (Supplementary Figure S2), which was only slightly lower with respect to the original scFvB1 (Kd = 3.49 × 10^−8^ M, [24]).

**Figure 3:**
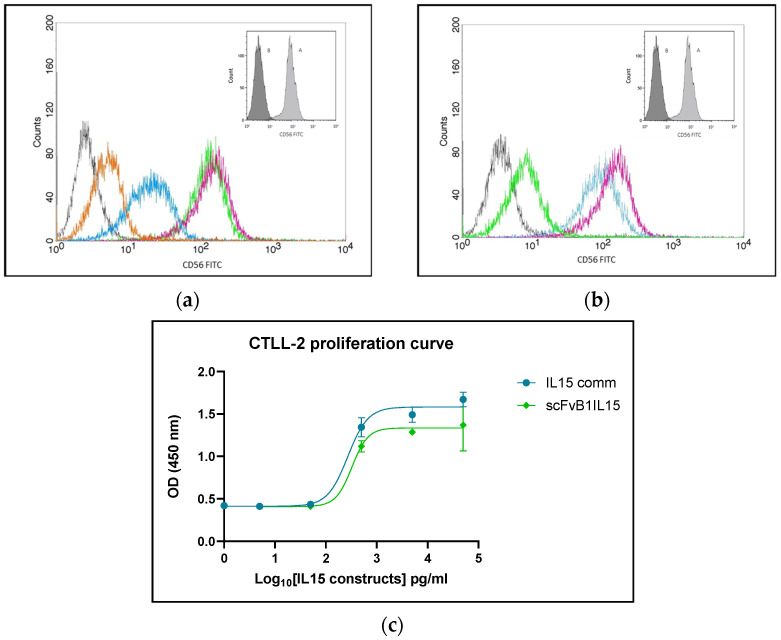
ScFvB1IL15 functional characterization. (**a**) ScFvB1IL15 binding titration. Flow cytometry of scFvB1IL15 at 20 µg/mL (violet), 1 µg/mL (green), 0.1 µg/mL (blue), and 0.01 µg/mL (orange) on NK92 cells. As a negative control, NK92 were marked with the mouse anti-hIL15 and FITC-conjugated anti-mouse secondary antibodies (black). In the box, NK92 labeling with commercial anti-CD56 FITC-conjugated antibody is depicted as the positive control (A—CD56 FITC staining; B—isotype). (**b**) ScFvB1IL15 binding specificity: results from a competition assay between scFvB1IL15 and scFvB1 or the irrelevant scFvGO for binding to NK92 cells. scFvB1IL15 at 1 µg/mL alone (violet); scFvB1IL15 at 1 µg/mL plus scFvGO at 250 µg/mL (blue); scFvB1IL15 at 1 µg/mL plus scFvB1 at 250 µg/mL (green); anti-hIL15 and FITC-conjugated anti-mouse secondary antibodies (black). Anti-IL15 monoclonal antibody was used to reveal the specific scFvB1IL15 signal. In the box, NK92 labeling with commercial anti-CD56 FITC antibody is depicted as the positive control (A—CD56 FITC staining; B—isotype). (**c**) scFvB1IL15 EC50 in CTLL-2 cell proliferation: OD results from a representative proliferation curve experiment with CTLL-2 cells treated with the commercial IL15 and scFvB1IL15 at 50, 5, 0.5, 0.05, and 0.005 ng/mL. Cell viability was measured using a Premix WST-1 colorimetric reagent. The points represent the averages of three replicates and the bars represents the SEMs. The GraphPad Prism 10.2.2 program was used to graph the data, extrapolate the non-fit regression curves, and calculate the EC50 values.

ScFvB1IL15-specific binding was further evaluated using flow cytometry assays on CD56+ NK92 cells. First, we performed dose–response binding experiment at four different concentrations of scFvB1IL15 (20, 1, 0.1, and 0.01 μg/mL) (Figure 3a).

Subsequently, we performed a competition assay via flow cytometry: 1 μg/mL of scFvB1IL15 was incubated alone or in the presence of scFvB1 or the irrelevant scFvGO at saturating concentration. As shown in Figure 3b, only scFvB1 induced a significant decrease in the binding of scFvB1IL15 to NK92 cells, while scFvGO did not. The small shift observed due to competition with scFvGO was likely because of steric hindrance as a large quantity of scFvGO was used. The residual binding after ScFvB1 competition was likely due to the binding of the IL15 arm to its receptor on the NK92 cells.

The IL15 moiety of the scFvB1IL15 immunocytokine was characterized by verifying the ability to induce proliferation in IL2-dependent CTLL-2 cells and comparing the EC50 value to that of the commercial purified IL15 (Figure 3c). No significant difference was observed between the EC50 values of the commercial IL15 and scFvB1IL15 (IL15 EC50: 0.283 ± 0.013 ng/mL; scFvB1IL15 EC50: 0.179 ± 0.075 ng/mL), with *p* = 0.564 (*p*-value was calculated using the Mann–Whitney test with three commercial IL15 curve replicates and three different scFvB1IL15 productions curves). The fact that the EC50 values of scFvB1IL15 and free IL15 were comparable (also considering that the concentration of scFvB1IL15 was calculated based on the IL15-targeted sandwich ELISA) is very important: in this way, the results of the functional experiments described later in this paper provided the right weight for the scFvB1-mediated targeting of NK cells to determine the immunocytokine effects after treating the PBMC samples.

### 3.3. ScFvB1IL5 Induces Specific Activation of NK Cells in PBMC Isolated from HDs

After the molecular characterization of scFvB1IL15, we proceeded to test the ability of the immunocytokine to recognize and activate the NK subpopulation in the PBMCs isolated from the HDs.

**Figure 4:**
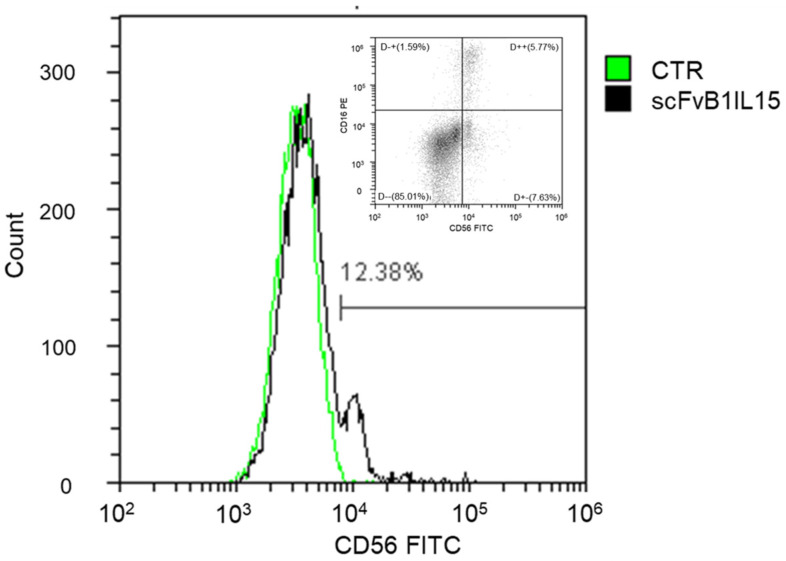
scFvB1IL15 binding to the NK population in a PBMC sample obtained from one representative HD out of the three tested. Control (CTR) is the binding profile of the anti-HIS and anti-mouse FITC-conjugated secondary antibodies. In the box at the top right, the same sample is marked with anti-CD16 PE and anti-CD56 FITC-conjugated commercial antibodies in order to compare the NK percentages.

Firstly, we evaluated its binding to the PBMCs from the HDs. scFvB1IL15 was able to recognize the whole NK population present in the PBMC samples isolated from the HDs. In fact, the percentage of PBMCs labeled with scFvB1IL15 (12.38%) corresponds exactly to the percentage of NK CD56-positive cells (both CD16 positive and CD16 negative, 13.4%) detected using the commercial anti-CD56 antibody in a representative HD, as shown in Figure 4. Supplementary Figure S3 shows the gating strategy for the identification of marked NK cells.

As further proof of the specific targeting of CD56-positive cells in the PBMC population, when we labeled the PBMCs from both the HDs and CLL patients treated ON with scFvB1IL15 and the commercial anti-CD56, we found that the CD56-positive population was almost completely lost. This differed from what was observed in the non-treated or IL15-treated PBMCs, suggesting that the binding of scFvB1IL15 to NK cells prevents the binding of the commercial anti-CD56 antibody (Supplementary Figures S4 and S5). This is why, during the functional assays described below, NK population gating was performed excluding CD14/CD19/CD3+ cells and not through positive labeling.

Next, we evaluated the impact of the scFvB1IL15 treatment specifically on NK cells by measuring cytokine production, degranulation marker expression, and direct cytotoxicity. All experiments were performed with whole PBMC and not with purified NK cells, since only with a mixed population is it possible to establish the role of scFvB1-mediated targeting. In fact, during a setting cytotoxicity experiment we conducted with two different HDs, we observed that the cytotoxicity curves relative to IL15 and scFvB1IL15 treatments were superimposable when purified NK cells were used as an effector population (indeed, at some points, IL15’s activity seems superior to that of scFvB1IL15), while they also separated with PBMCs at lower E:T ratios (Supplementary Figure S6).

To measure cytokine production and degranulation marker expression, we co-cultured PBMCs isolated from seven HDs with K562 cells, alone or in the presence of 10 ng/mL of the commercial IL15 or the scFvB1IL15 immunocytokine, and then we evaluated the intracellular IFNγ, MIP-1β, TNFα, and CD107a levels.

**Figure 5:**
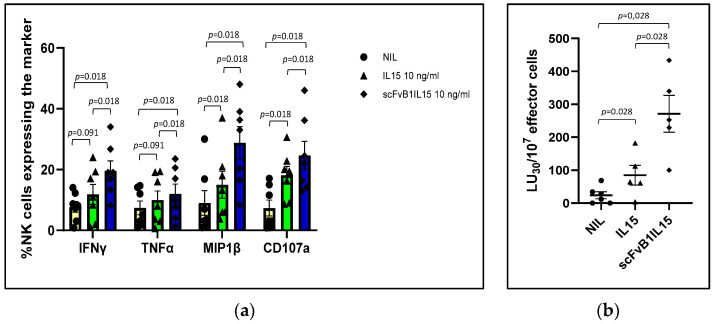
scFvB1IL15 functionality evaluation using PBMCs from the HDs. (**a**) NK intracellular cytokine and degranulation marker evaluation. The graph shows the mean percentages of the NK population positive for IFNγ, MIP-1β, TNFα, or CD107a after intracellular staining. The bars represent the SEMs. PBMCs from seven HDs were activated with IL15 or scFvB1IL15 at 10 ng/mL. *p* = significance level calculated with the Wilcoxon test. NIL: non-activated PBMCs. (**b**) NK lytic activity evaluation. The graph shows the NK-mediated cytotoxicity expressed as the number of lytic units (number of NK effector cells) contained in 10^7^ effector cells required to lyse 30% of K562 target cells, or LU_30_/10^7^. PBMCs were treated with 10 ng/mL of IL15 or scFvB1IL15. The averages of five independent HDs are presented, and the bars represent the SEMs. *p* = significance level calculated with the Wilcoxon test. NIL: non-activated PBMCs. The gating strategy and plots from a representative HD are shown in Supplementary Figure S7a,b.

As depicted in Figure 5a, scFvB1IL15 induced significant (*p* = 0.018) increases in IFNγ (19.61 ± 3.21%), TNFα (12 ± 3.21%), and MIP-1β (28.8 ± 5.31%) production, as well as increased CD107a (24.62 ± 4.65%) expression, in the NK cells subpopulation in comparison to IL15 (11.82 ± 3.30%, 9.95 ± 3.04%, 14.97 ± 4.42%, and 18.10 ± 2.93%, respectively), suggesting the real targeting of IL15 to NK cells that is mediated by scFvB1 when they are present in a moderate percentage in a mixed population with PBMCs.

To measure if the NK activation induced by scFvB1IL15 also resulted in a higher cytotoxic activity against the NK target cells (K562), we performed a cytotoxicity test with the PBMCs isolated from five HDs and co-cultured with K562 cells alone or in the presence of 10 ng/mL of the commercial IL15 or the scFvB1IL15 immunocytokine.

Cytotoxicity activity was calculated as lytic activity, defined as the number of lytic units (number of NK effector cells) contained in 10^7^ effector cells required to lyse 30% of the target cells, or LU_30_/10^7^.

As shown in Figure 5b, the lytic activity obtained with scfvB1IL15 was significantly higher than that of the IL15 treatments (271.12 ± 50.98 LU_30_/10^7^ effector cells for scFvB1IL15 versus 84.79 ± 9.15 LU_30_/10^7^ effector cells for free IL15, *p* = 0.028), confirming what we observed regarding cytokine production.

### 3.4. PBMCs from CLL Patients Exhibit Diminished NK-Mediated Direct Cytotoxicity and NKG2D Expression on NK Cell Surface

First, we analyzed NK cells obtained from 12 CLL patients, whose clinical and biological information is represented in Table 1, in order to characterize their dysfunction.

**Figure 6:**
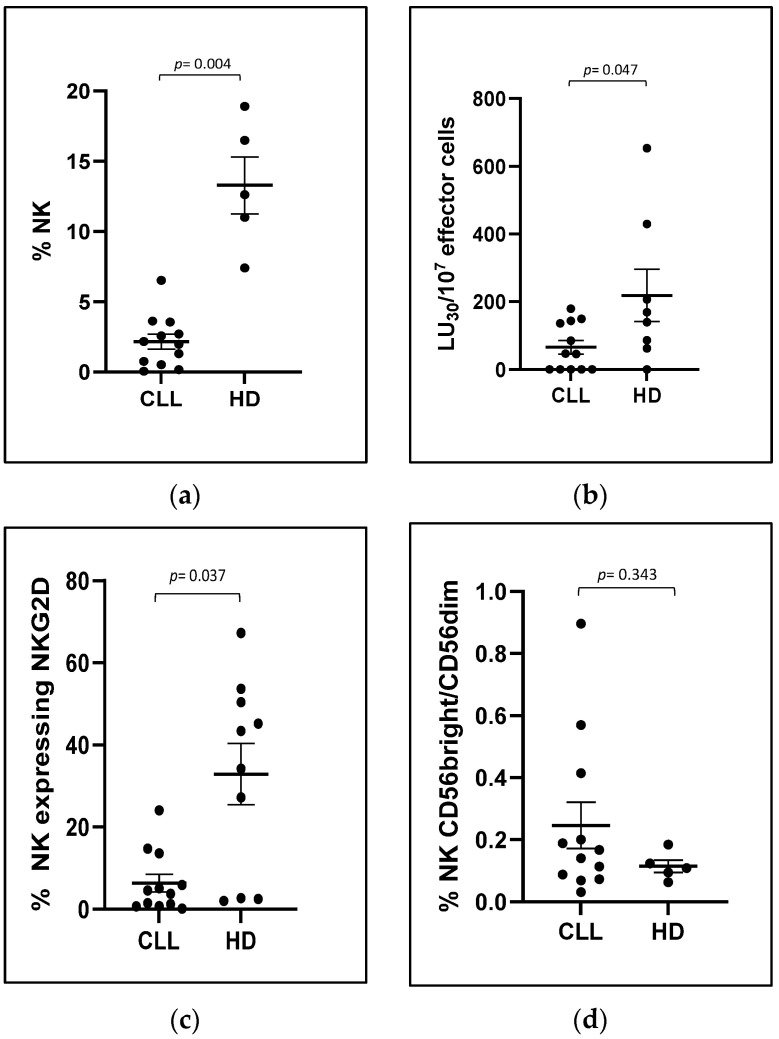
Differences between CLL patients and HDs. The graphs show the averages and SEMs of the experiments performed on PBMCs isolated from CLL patients (CLL) and HDs. The Mann–Whitney test was used to calculate the significance level of differences between the groups. (**a**) NK percentages in isolated PBMCs (12 CLL patients and 5 HDs). (**b**) Lytic activity (LU_30_/10^7^) of NK cells (effector cells) against K562 (target cells) (12 CLL patients and 8 HDs). The effective number of NK cells in each well, and the consequent real E:T ratio, was calculated by multiplying the number of PBMCs in each well by the known percentage of NK cells for each patient. (**c**) Surface expression of NKG2D receptor on NK cells (12 CLL patients and 10 HDs). (**d**) Percentages of CD56bright and CD56dim NK cells (12 CLL patients and 5 HDs).

We found that the patients we analyzed had a significantly reduced percentage of circulating NK cells (2.16 ± 0.53% in CLL patients versus 13.28 ± 2.02% in HDs, *p* = 0.004, Figure 6a), which was clearly due to the expansion of leukemic cells and a significant reduction in NK direct cytotoxicity, measured as the lytic activity required to kill 30% of K562 cells, the NK target cells (65.56 ± 21.07 LU_30_/10^7^ effectors cells in CLL patients versus 218.44 ± 77.06 LU_30_/10^7^ effectors cells in HDs, *p* = 0.047, Figure 6b).

Further, we observed a concurrent significant reduction in the expression of the activating receptor NKG2D on the surface of NK cells obtained from the CLL patients (6.92 ± 2.6% of NK cells expressing NKG2D in CLL patients versus 27.26 ± 7.11% in HDs, *p* = 0.037, Figure 6c). Meanwhile, there was a slight increase in the more immunomodulant and less cytotoxic CD56bright NK subpopulation, although it was not statistically significant (17.14 ± 3.9% in CLL patients versus 10.16 ± 1.58% in HDs, Figure 6d). No difference was observed in terms of production of cytokines and degranulation marker expression, nor in the expression of other activating receptors (Supplementary Figure S8).

### 3.5. ScFvB1IL15 Is Able to Stimulate Circulating NK Cells in PBMC Isolated from CLL Patients

To evaluate whether the scFvB1IL15 immunocytokine was also able to specifically stimulate NK cells in the PBMCs isolated from the CLL patients and restore the observed functional gaps, we first performed the cytotoxic assay as previously described for the samples from the HDs by using the equimolar concentration of IL15.

**Figure 7:**
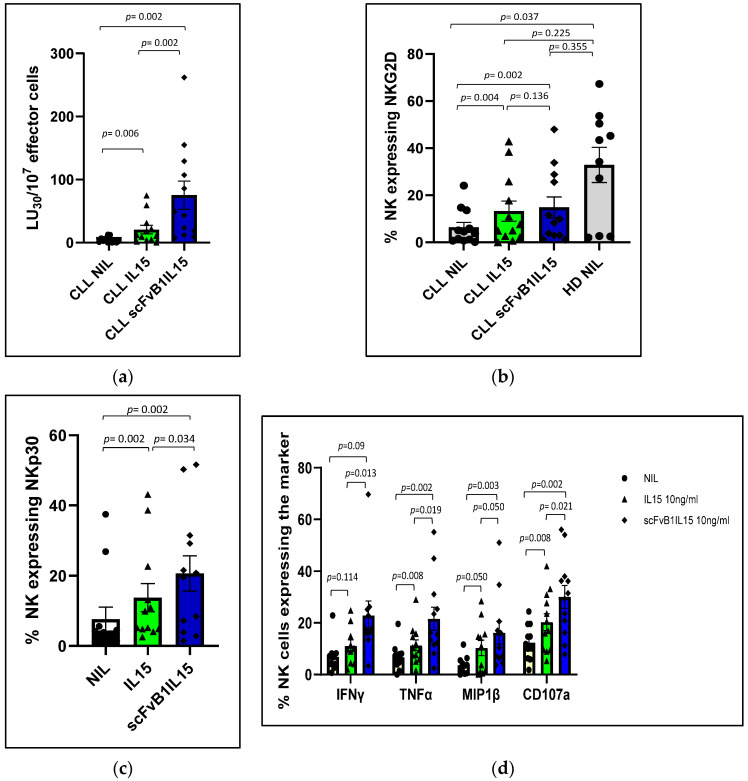
scFvB1IL15 functionality in PBMCs from the CLL patients. (**a**) Cytotoxic activity of NK cells in PBMC isolated from the CLL patients against K562 target cells: the graph shows the NK-mediated cytotoxicity of PBMCs expressed as the number of lytic units contained in 10^7^ effector cells (PBMCs) required to lyse 30% of K562 target cells, or LU_30_/10^7^. PBMCs from the CLL patients were not treated (NIL) or treated with 10 ng/mL of IL15 or scFvB1IL15. The averages of 12 CLL patients and the standard errors are presented. *p* = significance level calculated with the Wilcoxon test for comparisons among treatments. (**b**) NKG2D-activating receptor expression evaluation and (**c**) NKp30-activating receptor expression evaluation. The graphs (**b**,**c**) show the percentages of NK cells expressing the activating receptors in the PBMCs from CLL patients not treated (NIL) or treated with IL15 or scFvB1IL15 at 10 ng/mL. NIL HD is shown for NKG2D to evaluate the recovery of the expression of this receptor. The averages of 12 CLL patients and 10 independent HDs and the standard errors are presented. *p* = significance level calculated with Wilcoxon test for comparisons among treatments and with Mann–Whitney test for comparisons between patients and HDs. The gating strategy and plots from a representative patient are shown in Supplementary Figure S10. (**d**) CLL NK intracellular cytokine and degranulation marker evaluation. The graphs show the percentages of the NK population positive for IFNγ, MIP-1β, TNFα, or CD107a after intracellular staining. PBMCs from the CLL patients were not treated (NIL) or treated with IL15 or scFvB1IL15 at 10 ng/mL. The averages of 12 CLL patients are presented. The bars represent the SEMs. *p* = significance level calculated with Wilcoxon test. The gating strategy and plots from a representative patient are shown in Supplementary Figure S11.

We found that the IL15 treatment was able to increase the NK-mediated cytotoxicity in PBMCs from CLL patients (21.26 ± 7.2 LU_30_/10^7^ effector cells for PBMCs from CLL patients treated with IL15 versus 4.3 ± 1.31 LU_30_/10^7^ effector cells for non-treated PBMCs from HDs, *p* = 0.006), but scFvB1IL15 was able to induce an increase in cytotoxicity that was about 4-fold higher than the cytotoxicity induced by IL15 alone (80.77 ± 23.86 LU_30_/10^7^ effector cells for PBMCs from CLL patients treated with scFvB1IL15 versus 21.26 ± 7.2 LU_30_/10^7^ effector cells for PBMCs from CLL patients treated with IL15, *p* = 0.002; Figure 7a). Interestingly, Figure S9a,b show how the significant increase in cytotoxicity after the treatment with IL15 and scFvB1IL15 at different E:T ratios, in comparison to the non-treated PBMCs, is not accompanied by an increase in NK percentages. In the same way, although both IL15 and scFvB1IL15 were able to restore the NK-mediated cytotoxicity to the HD levels, scFvB1IL15 was able to better overcome the NK cell dysfunctionality. The graph in Figure S9c shows the comparison of NK-mediated cytotoxicity levels between PBMC from CLL treated with IL15 or scFvB1IL15 and non-treated PBMCs from HDs, taking into account the percentages of NK cells in each sample (respectively, 426 ± 99.9 LU_30_/10^7^ effector cells for PBMCs from CLL patients treated with IL15 and 2026.72 ± 414.92 LU_30_/10^7^ effector cells for PBMCs from CLL patients treated with scFvB1IL15 versus 218.44 ± 77.06 LU_30_/10^7^ effector cells for non-treated PBMCs from HDs).

**Figure 8:**
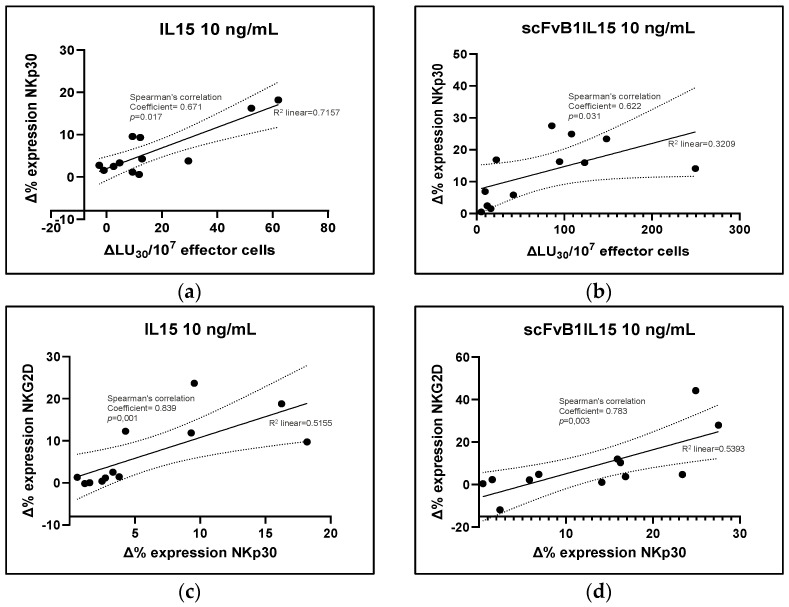
Increase in NKp30 expression contributes to direct cytotoxicity enhancement. (**a**,**b**) The graphs show the direct correlation between the variation in NKp30 expression and the variation in the direct cytotoxicity of NK cells expressed as the number of lytic units (number of NK effector cells) contained in 10^7^ effector cells required to lyse 30% of K562 target cells, or LU_30_/10^7^. The data refers to PBMCs from 12 CLL patients treated with 10 ng/mL of IL15 (**a**) or scFvB1IL15 (**b**). (**c**,**d**) The graphs show the direct correlation between the variation in NKp30 expression and the variation in NKG2D expression. The data refer to PBMCs from 12 CLL patients treated with 10 ng/mL of IL15 (**c**) or scFvB1IL15 (**d**). (Δ) = variation in the measured values after in vitro treatment; *p* = significance level calculated with Spearman’s non-parametric correlation test.

To verify whether the recovery of direct cytotoxicity was linked to the increase in NKG2D expression on NK cell surface, we also measured the levels of NKG2D after stimulation with K562 and treatment with IL15 or scFvB1IL15. We found that both treatments were able to induce an increase in NKG2D expression (13.78 ± 4.48% of NKG2D-positive NK cells in CLL PBMCs treated with IL15 and 15.31 ± 4.65% in CLL PBMCs treated with scFvB1IL15 versus 6.50 ± 2.33% for non-treated CLL PBMCs, with *p* = 0.004 and *p* = 0.002, respectively), whereas the difference between the level of NKG2D expressed on NK cells in PBMCs isolated from the HDs and that expressed on the treated CLL NK cells was no longer statistically significant (*p* = 0.25 for CLL PBMCs treated with IL15 versus non-treated PBMCs, and *p* = 0.355 for CLL PBMCs treated with scFvB1IL15 versus non-treated PBMCs). However, the means of NKG2D expression in the treated CLL NK cells did not reach the levels of NKG2D expressed on NK cells from the HDs. This observation suggests that NKG2D expression was not completely restored; in addition, the difference between the scFvB1IL15 and IL15 treatments was not statistically significant (*p* = 0.136, Figure 7b). Treatment with IL15 or scFvB1IL15 was also able to significatively increase the levels of another NK-activating receptor, NKp30 (13.78 ± 3.99% of NKp30-positive NK cells in CLL PBMCs treated with IL15 and 20.69 ± 5.01% in CLL PBMCs treated with scFvB1IL15 versus 7.68 ± 3.39% for non-treated CLL PBMCs, *p* = 0.002). The difference between the two treatments was statistically significant (*p* = 0.034, Figure 7c). Interestingly, we found a direct correlation between the increase in NKp30 expression and the increase in cytotoxicity for both the IL15 and scFvB1IL15 treatments (Figure 8a,b), with Spearman’s correlation coefficients of 0.671 (*p* = 0.017) and 0.622 (*p* = 0.031), respectively. We also found a positive correlation between the increase in NKp30 expression and the increase in NKG2D expression for both the IL15 and scFvB1IL15 treatments (Figure 8c,d), with Spearman’s correlation coefficients of 0.839 (*p* = 0.001) and 0.783 (*p* = 0.003), respectively.

Finally, we measured the levels of intracellular IFNγ and TNFα cytokines, MIP-1β chemokine, and CD107a degranulation marker expressed by NK cells in the PBMCs from the CLL patients after exposure to K562 and treatment with IL15 or scFvB1IL15. We found that both treatments were able to induce an increase in IFNγ (11.13 ± 2.3% of IFNℽ-positive NK cells in CLL PBMCs treated with IL15 and 22.388 ± 5.59% in CLL PBMCs treated with scFvB1IL15 versus 6.93 ± 1.93% for non-treated CLL PBMCs, with *p* = 0.114 and *p* = 0.009, respectively), TNFα (11.33 ± 2.08% of TNFα-positive NK cells in CLL PBMCs treated with IL15 and 21.63 ± 4.47% in CLL PBMCs treated with scFvB1IL15 versus 6.73 ± 1.42% for non-treated CLL PBMCs, with *p* = 0.008 and *p* = 0.002, respectively) and MIP1β (10.36 ± 2.84% of MIP1β-positive NK cells in CLL PBMCs treated with IL15 and 16.25 ± 4.24% in CLL PBMCs treated with scFvB1IL15 versus 3.6 ± 0.97% for non-treated CLL PBMCs, with *p* = 0.050 and *p* = 0.003, respectively). In the same way, we found an increase in CD107a expression for both treatments (20.35 ± 3.04% of CD107a-positive NK cells in CLL PBMCs treated with IL15 and 30.08 ± 4.53% in CLL PBMCs treated with scFvB1IL15 versus 12.42 ± 1.94% for non-treated CLL PBMCs, with *p* = 0.008 and *p* = 0.002, respectively). Notably, for all intracellular markers, we observed that the scFvB1IL15 treatment exerted a statistically significant stronger effect compared to free-IL15 treatment (*p* = 0.013 for IFNγ, *p* = 0.019 for TNFα, *p* = 0.050 for MIP1β, and *p* = 0.021 for CD107a; Figure 7d).

Taken together, our results suggest that, due to the antibody arm, treatment of the CLL patient-derived PBMCs with scFvB1IL15 enhanced the effects of IL15 on the CD56-positive subpopulation, thus potentiating the effects of free IL15 and resulting in the specific stimulation of NK cells.

## 4. Discussion

Herein, we describe the construction and functional characterization of an immunocytokine, scFvB1IL15, designed to target NK cells through the CD56-specific scFvB1 antibody arm and activate the IL15 receptor on the same cell, with the aim of inducing targeted—and, therefore, improved—NK stimulation, as compared to free IL15.

Our results confirm that the antibody arm has the right affinity and specificity to target NK cells in a mixed population of cells, such as PBMCs, even when NK cells are present in a small percentage, as in patients with progressive CLL.

As expected, the immunocytokine was also able to activate NK cells from the HD PBMCs better than free IL15 in terms of both direct cytotoxicity and production of cytokines and chemokines, thus underlining its potential for enhancing a receptor-mediated biological action in a particular cell subpopulation. Interestingly, when purified NK cells were used in cytotoxicity assays, the difference between the immunocytokine and the free IL15 was cancelled, since in this case no targeting effect was necessary, as there was no mixed population (Supplementary Figure S6). This fact further underlines how the enhancement of IL15 activity was not due to new or stronger functions acquired thanks to IL15 conjugation to the antibody in itself but only to a targeting effect on the specific subpopulation.

The same potentiated effect was observed on PBMCs derived from CLL patients, which, in line with previously published data, showed a dysfunctionality of CLL-NK cells with a reduction in NK-mediated direct cytotoxicity (specifically measured with K562 target population) and expression of the activating receptor NKG2D in NK cells.

In particular, when PBMCs from patients with progressive CLL were cultured with IL15 or scFvB1IL15, we observed the restoration of the direct NK cytotoxic activity to HDs levels (even exceeding them) in both conditions; however, the scFvB1IL15 immunocytokine was more highly effective in increasing the NK-mediated direct cytotoxicity in CLL patients. This observation suggests that the restoration of NK-mediated cytotoxicity in CLL patients might be obtained using a lower concentration of conjugated IL15, thus further reducing off-target effects.

The increase in cytotoxicity to HD levels (calculated considering the effective number of NK cells and E:T in the well) was not due to an NK proliferation (Supplementary Figure S9b,c) but to a real restoration of NK cells functions.

In our experiment setting, one factor contributing to IL15-mediated restoration was the cumulative increase in NK-activating receptors. Specifically, we observed a significant increase in NKG2D expression in the NK cells from CLL patients when their PBMCs were cultured with scFvB1IL15 or IL15 compared to the non-stimulated condition. This enhancement contributed to reducing the difference in NKGD expression between the PBMCs from the HDs and CLL patients, which was no longer statistically significant after treatment. However, higher levels of NKGD expression were maintained in the PBMCs from the HDs. Interestingly, at the same time, both IL15 and scFvB1IL15 were able to increase the expression of the NKp30 receptor, another key receptor participating in NK-mediated direct cytotoxicity [33,34]. The correlation between the increase in the NKp30 expression and the direct cytotoxicity percentages induced by the IL15 or scFvB1IL15 treatment suggests a contribution of the NKp30 receptor in the restoration of NK-mediated cytotoxicity, when the recovery of the NKG2D receptor after both the IL15 and scFvB1IL15 treatments was not complete when compared to the HD levels. This is an interesting finding, as NKG2D expression was impaired in the CLL patients compared to the HDs at baseline while NKp30 expression was not, suggesting that compensation among different NK receptors can take place in order to restore the impaired NK function.

Another IL15-mediated effect underlying the enhancement of CLL NK functionality was the increase in other factors such as the degranulation marker CD107a along with pro-inflammatory cytokines and chemokines, such as IFNℽ, TNFα, and MIP1β [35,36].

Clearly, other mechanisms and factors, in addition to those explored by us, may be involved in the ability of IL15 to restore NK cell functions; nevertheless, our observations show that IL15 and scFvB1IL15 have the same mode of action, but the effects induced by scFvB1IL15 were always statistically higher than those obtained with free IL-15.

It would be interesting to establish if the potentiated activity of scFvB1IL15 could also translate into overcoming the possible intrinsic resistance of CLL cells to NK-mediated direct killing [12,37]; a limitation of our study design is, in fact, the absence of experiments evaluating the effect of NK activation using leukemic cells as the target, leaving this aspect unexplored.

On the other hand, using autologous CLL cells as the target instead of the K562 cell line could have introduced high variability in the obtained cytotoxic assay outcomes. Moreover, considering the need to use total PBMCs to demonstrate the potency of scFvB1-mediated targeting, the use of K562 cells allowed us to investigate the specific activation and function of NK cells and to reduce the impact of CD8 T-cell-mediated direct cytotoxicity [38].

What we can affirm with our experiments is that the treatment with scFvB1IL15 seems to be able to overcome the immunosuppressive soluble factors that may be released by leukemic cells present in the cell cultures.

Further studies need to be carried out to evaluate the effect of the scFvB1IL15 treatment on the killing of CLL cells, alone or in combination with molecules that can overcome the intrinsic resistance of CLL cells to direct killing [36]. Moreover, the IL15 antibody-mediated stimulation of NK cells should be further investigated in the context of CLL pathology, including evaluation of whether leukemic cells are preserved by the IL15-mediated proliferating action [21], and in the context of CAR-NK cellular therapy for CLL.

Taken together, our results provide evidence for the ability of antibody-mediated targeting to maximize the activity of IL15 in a particular cell subset and for the efficacy of this approach in potentiating dysfunctional NK cells.

Overall, the scFvB1IL15 immunocytokine joins the group of IL15-containing con-structs developed to overcome the limitations of IL15 as a single immunotherapeutic agent that we previously mentioned in the Introduction section (Section 1); some of these constructs are being investigated in clinical trials or are in the preclinical stages [17,18,19,39]. Some constructs enhance the activity of IL15 via conjugation to IL15Rα, an-ti-PDL1/PD1 mAb construct, or NKG2D extracellular domain (N809, anti-PD1-IL15 mutein, and dsNKG2D-IL15). Multifunctional heteromeric fusion protein complexes (HFPCs, such as HCW) containing IL12, IL15, and IL18 have also been developed to exploit the synergy of cytokines. Other constructs (HcW9107, IL15 trike) direct the activity of IL15 specifically to NK cells via conjugation to an anti-CD16 scFv. Finally, another group of constructs (anti-GD2RLI, 2B8T2M, IL15 trike) exploits the targeting mediated by a specific antibody for a tumor-associated antigen (TSA) to direct the activity of effector cells activated by the IL15/IL15Rα sushi domain complex to tumor cells.

The scFvB1IL15 immunocytokine, described in this manuscript, directs IL15 to NK cells through specific binding to the CD56 antigen and restores the NK population in CLL patients to its physiological functions in a targeted way. In this regard, we cannot exclude that, in vivo, the immunocytokine can also act on the small subset of more cytotoxic CD8+ T and NKT cells which also express CD56 and IL15R [40,41], but considering the percentages of CD8+ CD56+ T cells (less than 5%) and of NKT cells (less than 1%) in peripheral blood, we can state that the targeting of IL15 by an anti-CD56 antibody can greatly reduce the binding of IL15 to the large variety of cells that express IL15R and are responsive to IL15.

Additionally, being antigen-independent, scFvB1IL15 could also be explored in other disorders that involve dysfunction of cytotoxic NK cells or in adoptive transfer protocols to improve and sustain NK cell expansion and activation.

## 5. Conclusions

In conclusion, the scFvB1IL15 construct demonstrates the feasibility of concentrating the action of a cytokine on a specific subpopulation, potentiating its activity, and shows promise as a tool that could contribute to anti-tumor activity and, hopefully, to successful NK cell-based immunotherapy.

## Figures and Tables

**Table 1:** Clinical and biological characteristics of CLL patients.

Characteristics	Mean	Number of Patients	Percentage	Range
**Age (years)**	63			40–78
	
**Sex**				
Male		6	50	
Female		6	50	
**RAI stage**				
0–II		6	50	
III–IV		6	50	
**BINET stage**				
A		0	0	
B		6	50	
C		6	50	
**IGHV**				
Unmutated		5	41.6	
Mutated		7	58.3	
**FISH**				
Del 13q		4	33.3	
Trisomy 12		4	33.3	
No aberrations		4	33.3	
TP53 deletion or mutation		none	0	
Lymphocyte count × 10^9^/L	83			13–192
Platelet count × 10^9^/L	130			78–201
Hb, g/dL	12			9.2–15
IgG, μγ/dL	778			310–1520
b2 microglobulin, mg/mL	3.75			1.7–5
LDH, mU/mL	204.25			125–278

## Data Availability

All primary data are available from the corresponding authors upon request.

## Supplementary Materials

The following supporting information can be downloaded at: https://www.mdpi.com/article/10.3390/biom15010117/s1, Figure S1: ScFvB1IL15 quantification using sandwich ELISA; Figure S2: Affinity calculation of scFvB1IL15; Figure S3: Gating strategy for identification of NK population; Figure S4: Negative and positive staining to identify NK population in the PBMCs of HDs; Figure S5: Negative and positive staining to identify NK population in PBMCs from CLL patients; Figure S6: Cytotoxic curves with whole PBMC and purified NK cells from HDs; Figure S7: Gating strategy for PBMCs from HDs; Figure S8: Comparison of the means of NK intracellular cytokines (IFNγ, TNFα, and MIP1β), CD107a, and activating receptors (NKp30 and NKp46) between the CLL patients and healthy donors; Figure S9: Cytotoxic curves in CLL patients; Figure S10: Gating strategy for PBMCs from CLL patients (receptors); Figure S11: Gating strategy for PBMCs from CLL patients (intracellular markers).
