# Supplementary material for: A New Antibody–Cytokine Construct Targeting Natural Killer Cells: An Immunotherapeutic Approach to Chronic Lymphocytic Leukemia
Source: Biomolecules. 2025 Jan 13;15(1):117. doi: 10.3390/biom15010117 (PMC11764099; doi:10.3390/biom15010117)
Supplement: Supplementary file 1 [file biomolecules-15-00117-s001.zip › biomolecules-3304384-supplementary.pdf]

Supplementary figures' legends

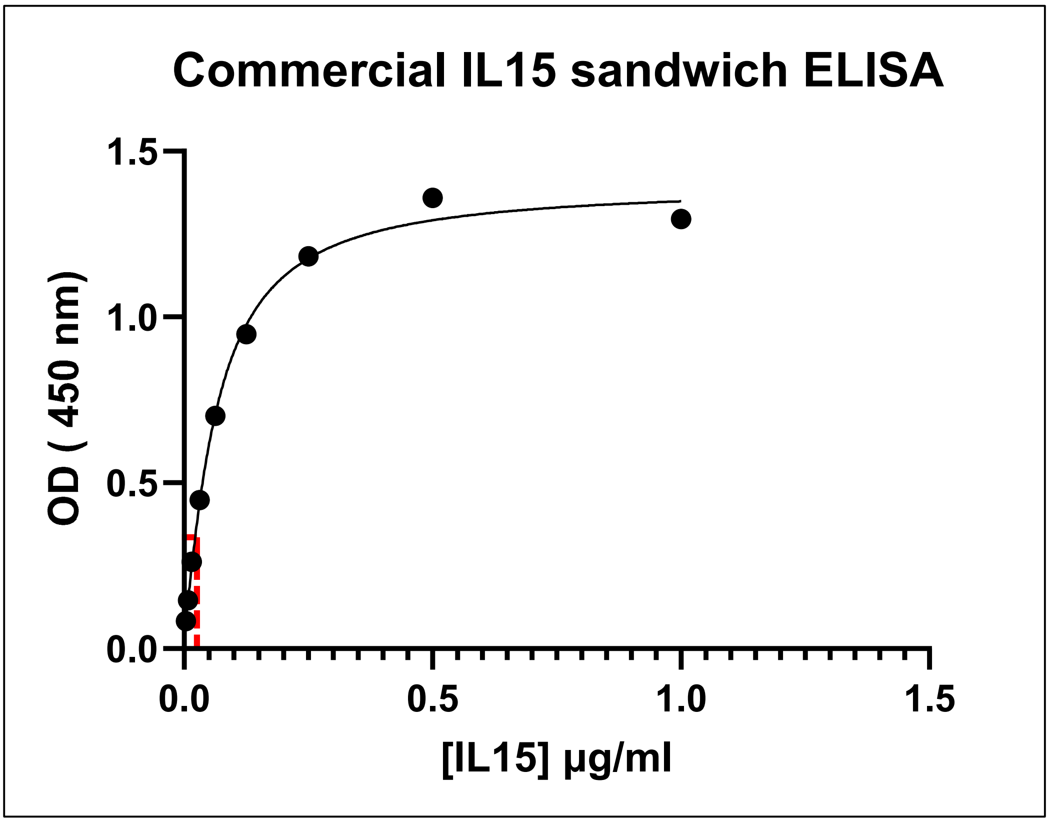

(A)

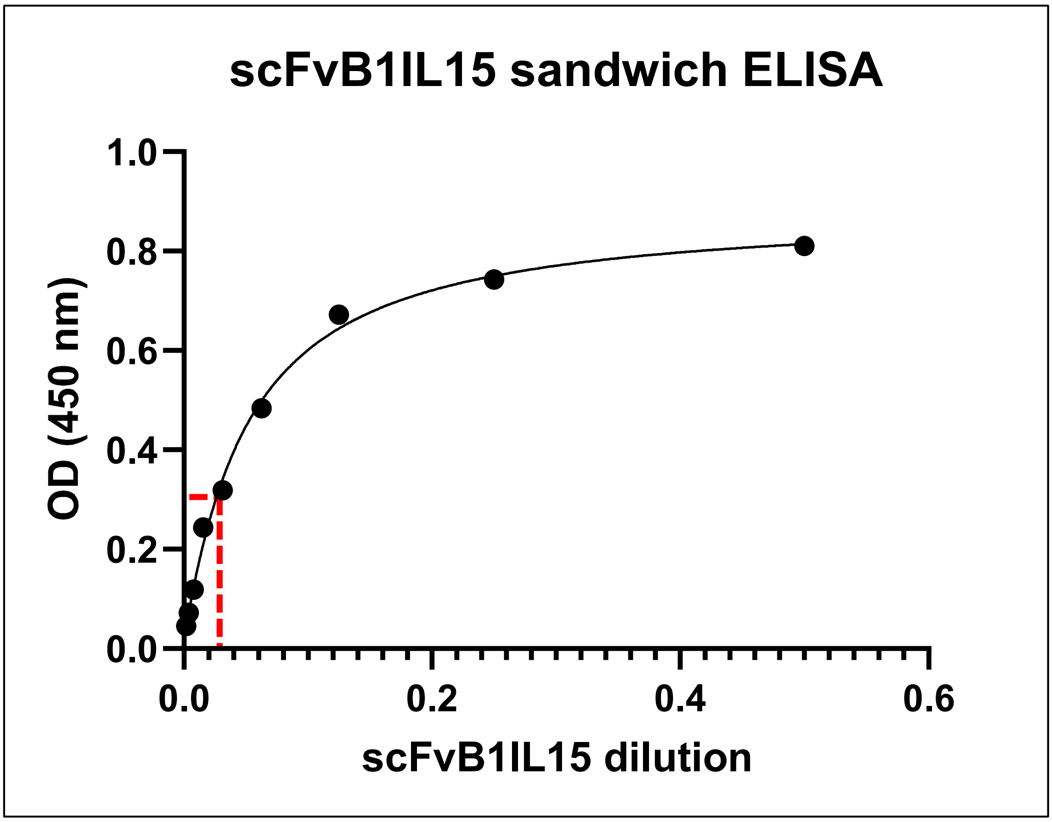

(B)

Supplementary Figure S1. ScFvB1IL15 quantification using sandwich ELISA: the plot on the left (A) shows the OD from the sandwich ELISA at different concentration of the commercial IL15. The plot on the right (B) shows the OD from the sandwich ELISA with different dilutions of the purified scFvB1IL15 protein. The GraphPad prism 10.2.2 program was used to graph the data, extrapolate a non-fit regression curve, and calculate the concentration of IL15 and the dilution of scFvB1IL15 at OD = 0.3 (chosen as a point in the linear part of both curves). ScFvB1IL15 concentration was calculated as the concentration of IL15 at the 0.3 OD divided by the scFvB1IL15 dilution at the same OD. Sandwich ELISA was performed by coating the wells of the plate with a monoclonal anti-IL15 antibody and detection was carried out using a streptavidin-conjugated polyclonal anti-IL15 antibody.

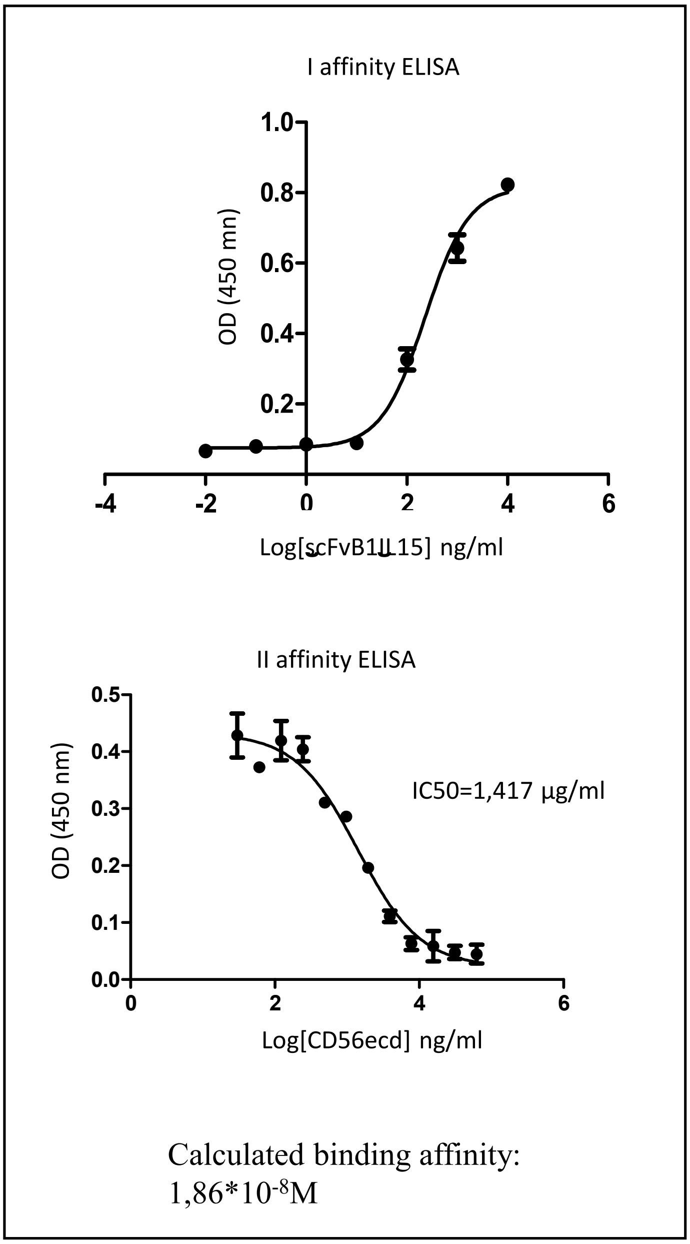

Supplementary Figure S2. Affinity calculation of scFvB1IL15. Affinity binding (expressed as the binding dissociation constant) was calculated using a two-step ELISA, as described in the Methods

section. The first ELISA step (top) was performed to determinate the immunocytokine concentration exhibiting 50% binding (EC50). The wells were coated with 0.5 ug of CD56 protein and then the serial dilutions of scFvB1IL15 (X-axis, ng/ml). The second ELISA step (bottom) was performed to determine the ratio of the concentration of free immunocytokine at equilibrium. The wells were coated with 0.5 ug of CD56 protein and then the serial dilutions of the same antigen (X-axis, ng/mL) were incubated in a solution with the scFvB1IL15 construct at the determined EC50 (calculated from the first ELISA step). The EC50 value from this second ELISA step was used to calculate the dissociation constant

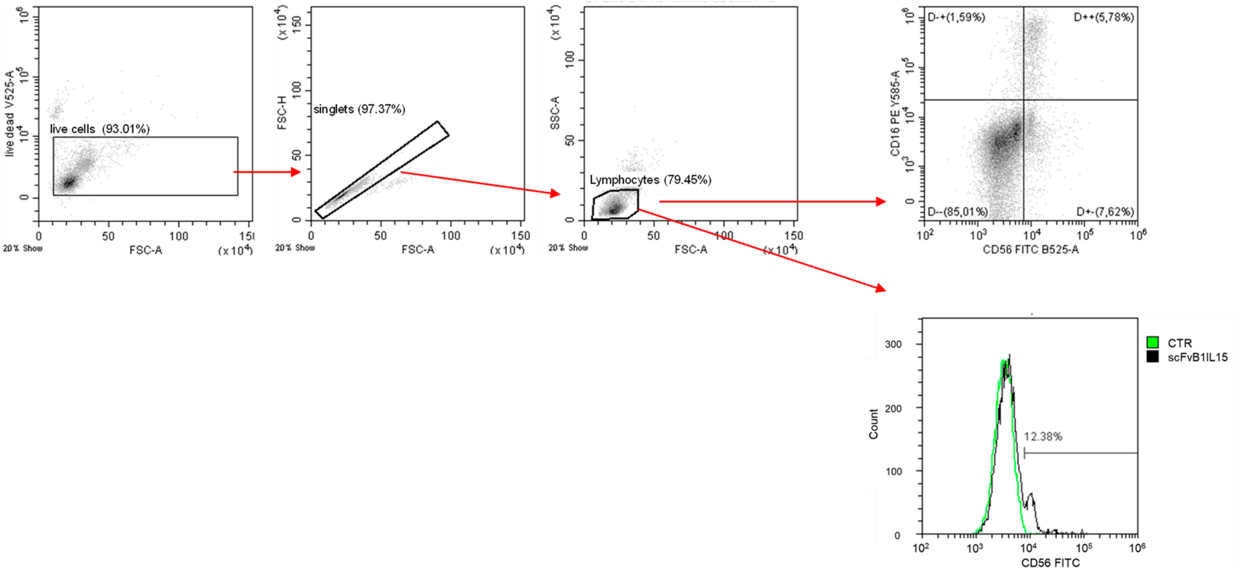

Supplementary Figure S3. Gating strategy for identification of NK population. Viable cells were gated via forward scatter (FCS) and exclusion of the LIVE/DEAD™ Fixable Aqua reagent, and then the singlets were gated; lymphocytes were identified based on the physical parameters, and in this final population, CD56-positive cells (both CD16 pos and CD16 neg) were identified. The antibodies used for this staining were Beckton Dickinson CD56 FITC (340410, clone NCAM16.2) and CD16 PE (561313).

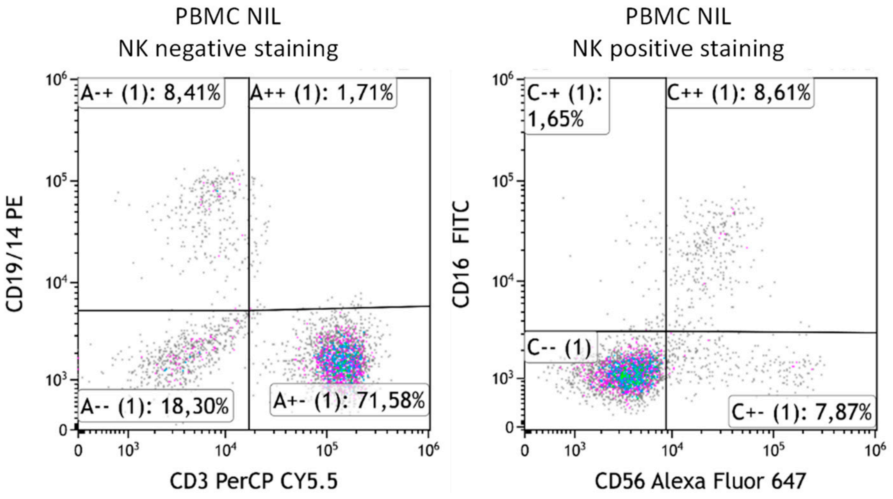
PBMC NIL
NK negative staining
A-+ (1): 8,41%
A++ (1): 1,71%
A-- (1): 18,30%
A+- (1): 71,58%
CD19/14 PE
CD3 PerCP CY5.5
PBMC NIL
NK positive staining
C-+ (1): 1,65%
C++ (1): 8,61%
C-- (1)
C+- (1): 7,87%
CD16 FITC
CD56 Alexa Fluor 647

(**A**)

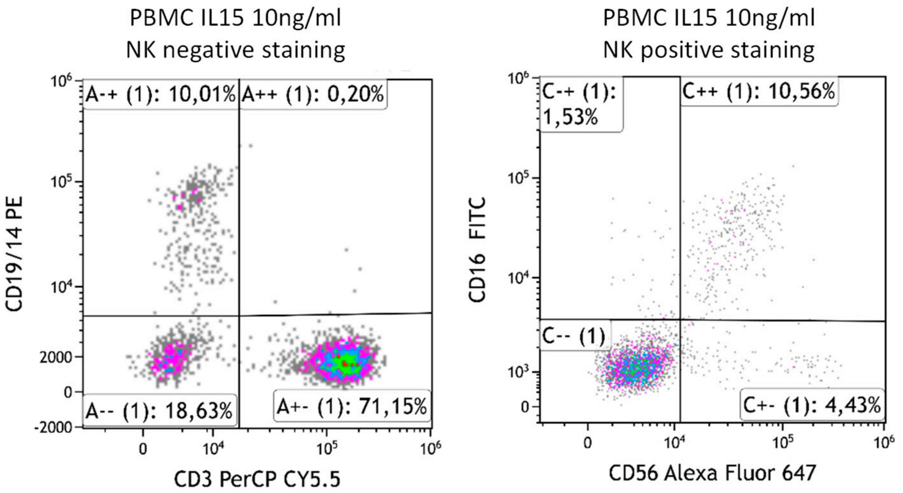
PBMC IL15 10ng/ml
NK negative staining
A-+ (1): 10,01%
A++ (1): 0,20%
A-- (1): 18,63%
A+- (1): 71,15%
CD19/14 PE
CD3 PerCP CY5.5
PBMC IL15 10ng/ml
NK positive staining
C-+ (1): 1,53%
C++ (1): 10,56%
C-- (1)
C+- (1): 4,43%
CD16 FITC
CD56 Alexa Fluor 647

(**B**)

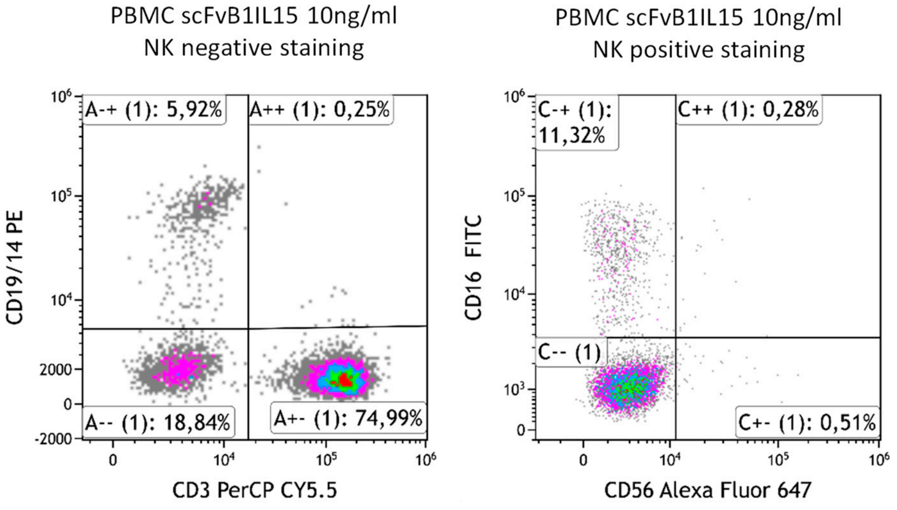
PBMC scFvB1IL15 10ng/ml
NK negative staining
A-+ (1): 5,92%
A++ (1): 0,25%
A-- (1): 18,84%
A+- (1): 74,99%
CD19/14 PE
CD3 PerCP CY5.5
PBMC scFvB1IL15 10ng/ml
NK positive staining
C-+ (1): 11,32%
C++ (1): 0,28%
C-- (1)
C+- (1): 0,51%
CD16 FITC
CD56 Alexa Fluor 647

(**C**)

Supplementary Figure S4. Negative and positive staining to identify NK population in the PBMCs of HDs. The figure shows the negative (CD19/CD14 PE negative and CD3 PerCP-Cy5.5 negative) and positive (CD56 Alexa Fluor 647 positive and CD16 FITC negative/positive) staining of NK cells in untreated (NIL) (A), IL15-treated (B), and scFvB1IL15-treated (C) PBMCs from a representative healthy donor.

Negative staining results in the same NK percentages across all three conditions; positive staining results the same NK percentages in the untreated and IL-5-treated PBMCs, while in the scFvB1IL15-treated PBMCs, the CD56 NK population is completely lost because of the receptor competition and all cells shift towards CD16-positive CD56 negative staining. The antibodies used for this staining were Beckton Dickinson anti-CD3 PerCP-Cy5.5 (560835), CD19PE (555413), CD14PE (555398), CD56 Alexa Fluor 647 (557711, clone B159), and CD16 FITC (561308).

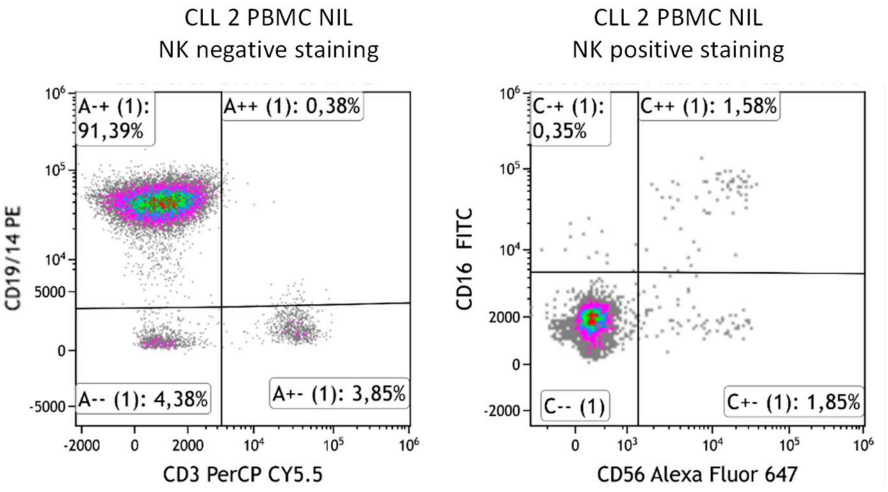

(A)

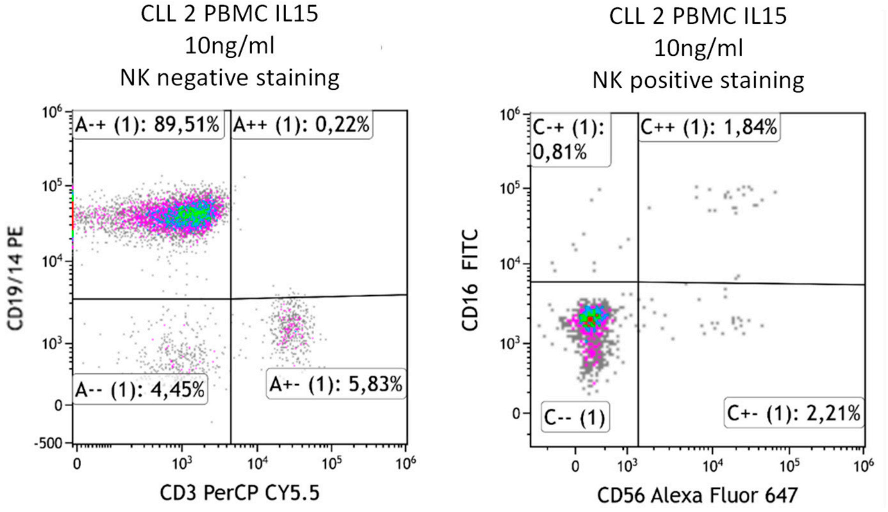

(B)

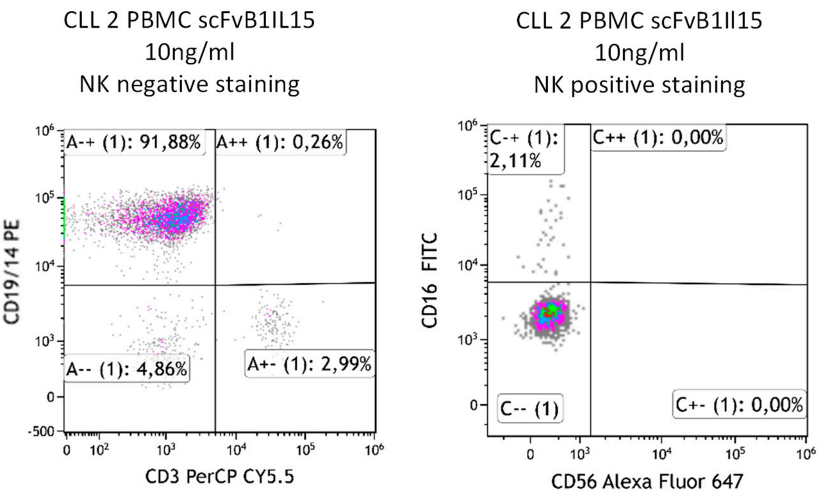

(**C**)

Supplementary Figure S5. Negative and positive staining to identify NK population in PBMCs from CLL patients. The figure shows negative (CD19/CD14 PE negative and CD3 PerCP-Cy5.5 negative) and positive (CD56 Alexa Fluor 647 positive and CD16 FITC negative/positive) staining of NK cells in untreated (NIL) (A), IL15-treated (B), and scFvB1IL15-treated (C) PBMCs from a representative CLL patient.

Negative staining results in the same NK percentages across all three conditions; positive staining results in the same NK percentages in untreated and IL-5-treated PBMCs, while in the scFvB1IL15-treated PBMCs, the CD56 NK population is completely lost because of the receptor competition and all cells shift towards CD16-positive CD56 negative staining. The antibodies used for this staining were Beckton Dickinson anti-CD3 PerCP-Cy5.5 (560835), CD19PE (555413), CD14PE (555398), CD56 Alexa Fluor 647 (557711, clone B159), and CD16 FITC (561308)

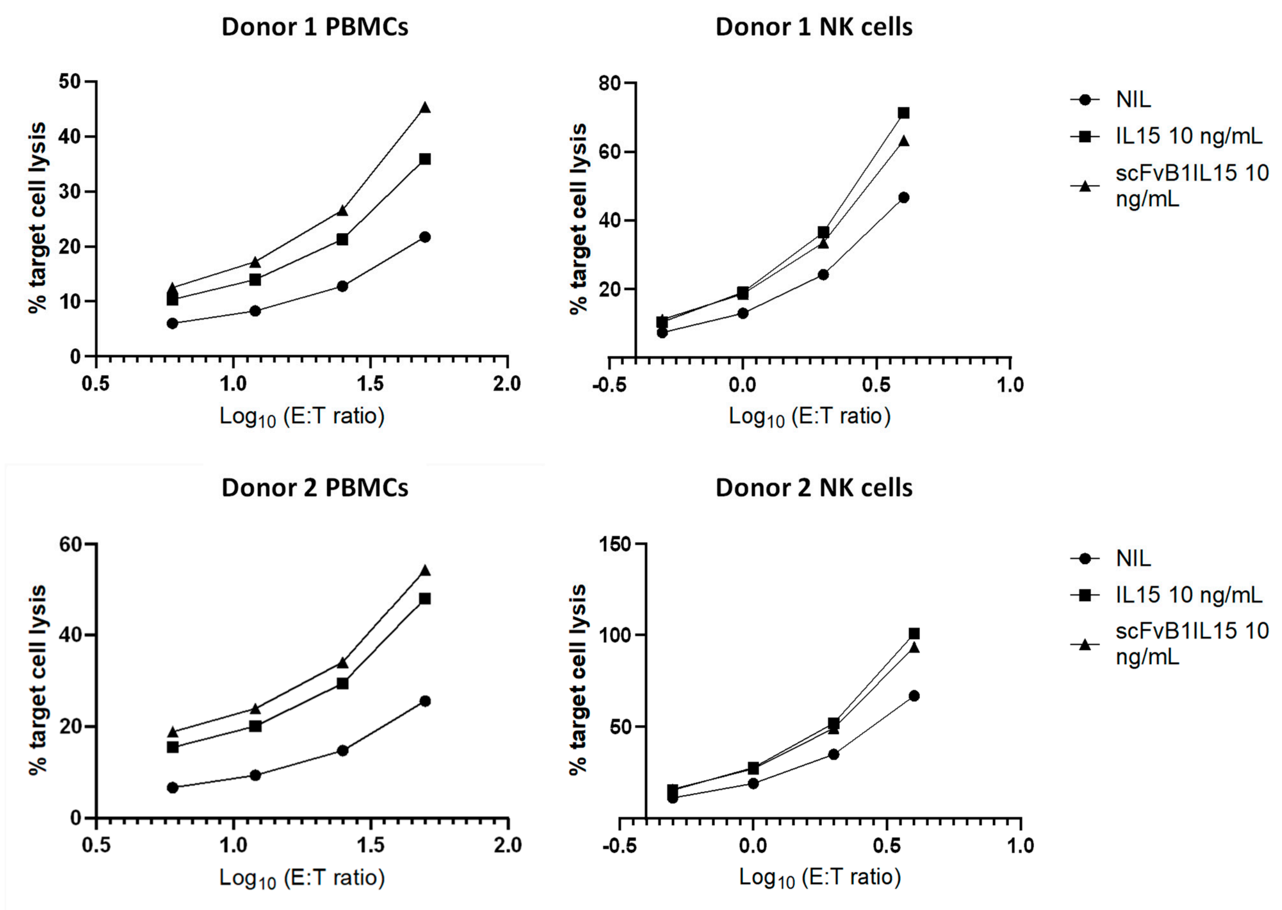

Supplementary Figure S6. Cytotoxic curves with whole PBMC and purified NK cells from HDs. The graphs show the percentages of K562 (T) lysis after co-culture with untreated (NIL) or treated (IL-15 or scFvB1IL15) whole PBMCs (left) and purified NK (right) cells from two healthy donors (donor 1 upper and donor 2 lower) at different E:T ratios. For PBMCs, E:T ratios were 50:1, 25:1, 12,5:, 6,25:1; for purified NK cells E:T ratios were 4:1, 2:1, 1:1, 0,5:1.

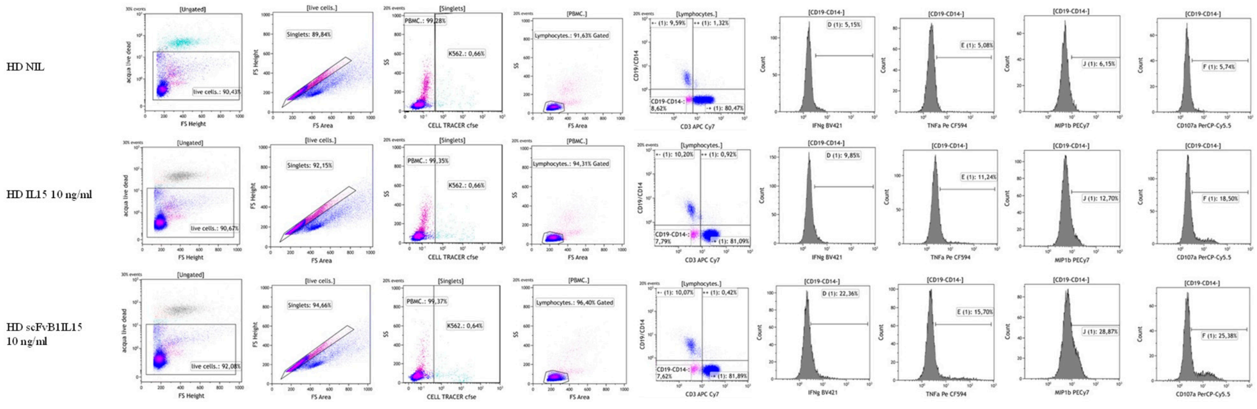

(A)

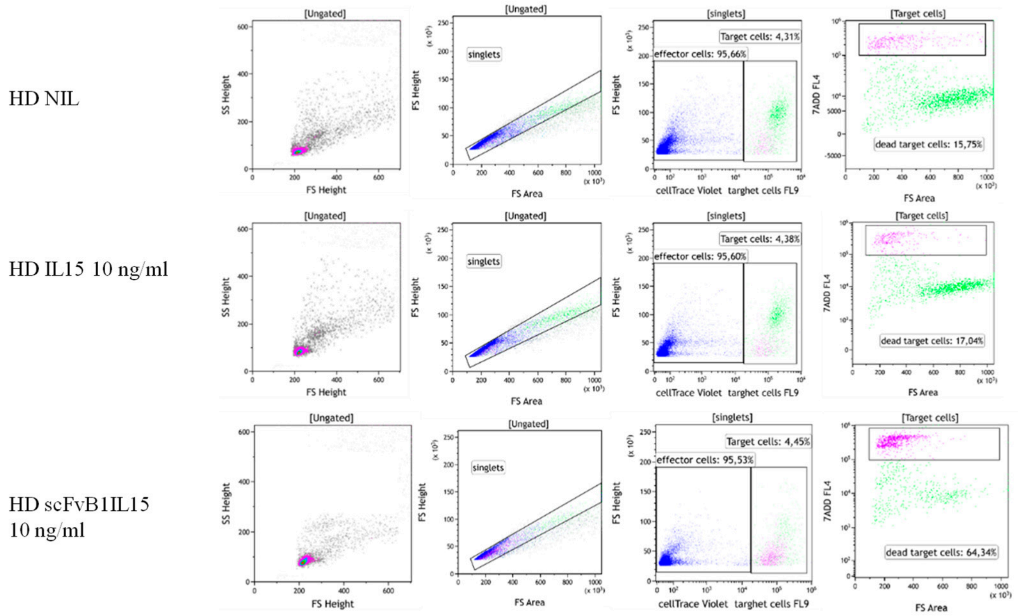

(B)

Supplementary Figure S7. Gating strategy for PBMCs from HDs. (A) Gating strategy for a representative HD for the comparison of intracellular cytokines and CD107a marker in untreated, IL15-treated, and scFvB1IL15-treated PBMCs. (B) Gating strategy for a representative HD for the evaluation of K562 lysis in non-treated, IL15-treated, and scFvB1IL15-treated PBMCs. In this figure, the nominal E:T = 25:1 condition is reported as an example.

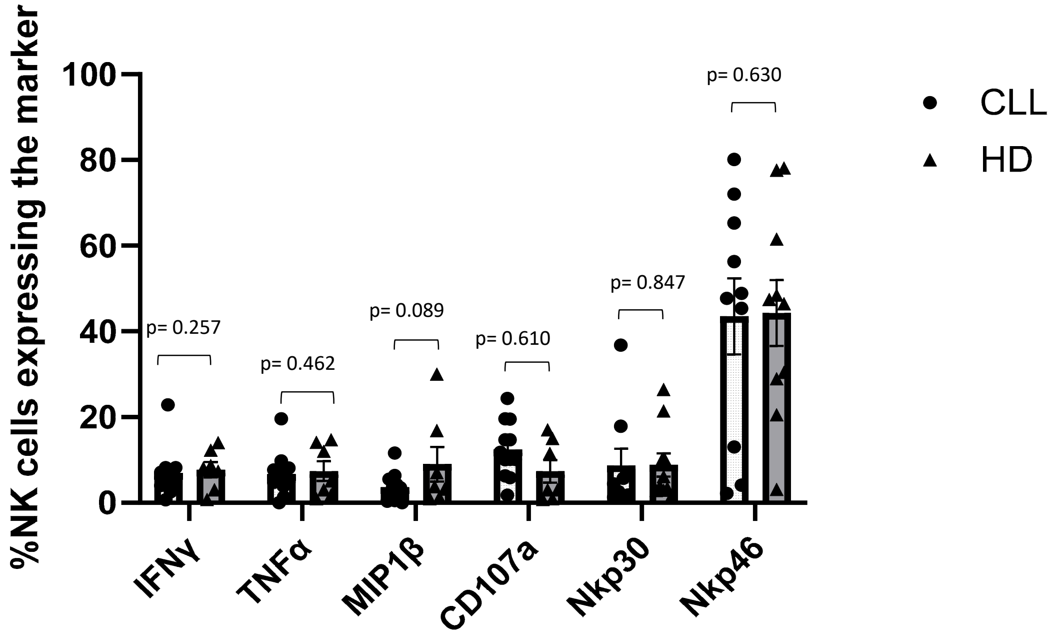

Supplementary Figure S8. Comparison of the means (bars represent the SEMs) of NK intracellular cytokines (IFNγ, TNFα, and MIP1β), CD107a, and activating receptors (NKp30 and NKp46)

between the CLL patients and healthy donors. Data are expressed as the averages of the experiments performed on the PBMCs isolated from 12 CLL patients and 7 healthy donors (HD) for intracellular markers and 10 HDs for activating receptors. The Mann–Whitney test was used to calculate the significance level of differences between the groups

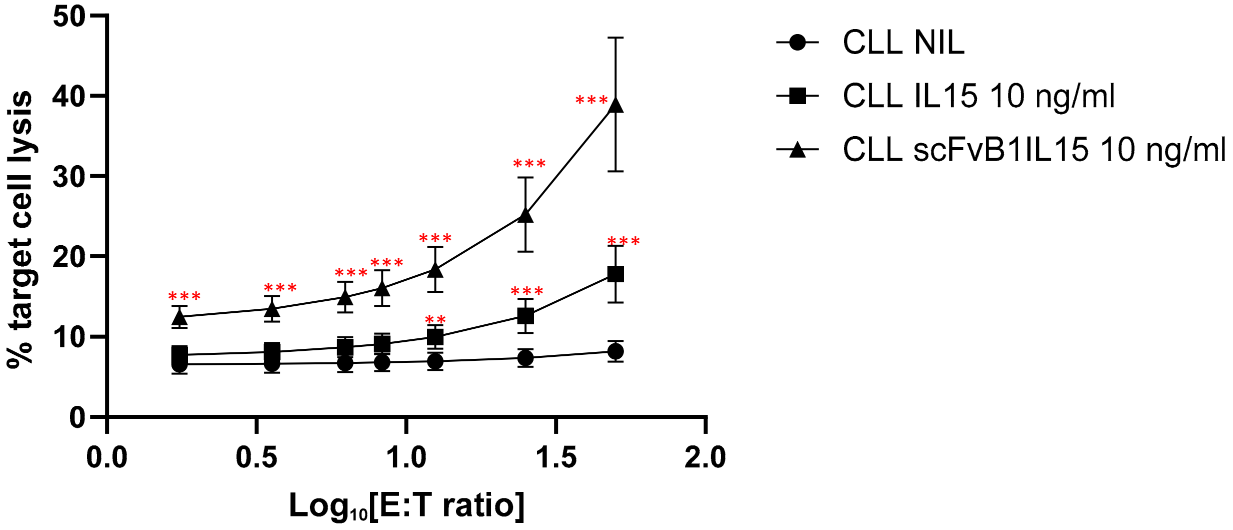

(A)

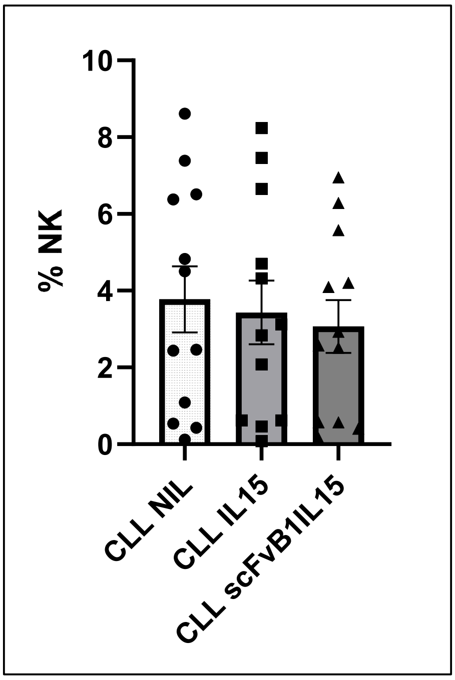

(B)

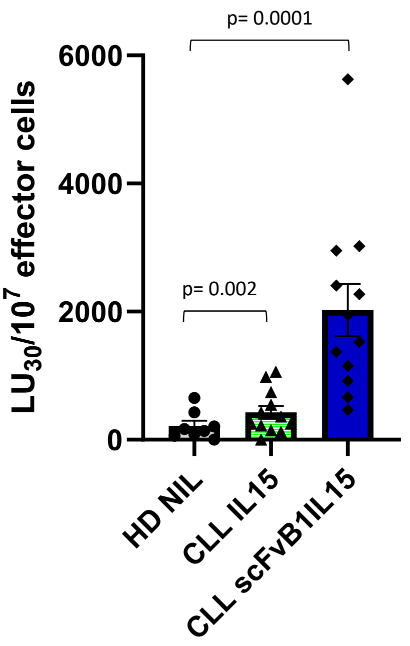

(**C**)

Supplementary Figure S9. Cytotoxic curves in CLL patients. (A) The graph shows the means of the percentages of K562 (T) lysis after co-culture with untreated (NIL) or treated (IL-15 or scFvB1IL15) PBMCs from 12 CLL patients (E) at different E:T ratios. The bars represent the SEMs. The Wilcoxon test for parametric values was used to calculate the significance level of differences between different conditions. ** $p < 0.01$; *** $p < 0.005$. (B) The graph shows the means of the percentages of NK cells in non-treated (NIL) and treated (IL15 or scFvB1IL15) PBMCs from 12 CLL patients after co-culture with K562. The bars represent the SEMs. (C) The graph shows the lytic activity ($LU_{30}/10^7$) of NK cells (effector cells) from CLL patients treated with IL15 or scFvB1IL15 against K562 (target cells) versus non-treated NK cells from HDs (12 CLL patients and 8 HDs). The effective number of NK cells in each well and the consequent real E:T ratio, was calculated by multiplying the number of PBMCs in each well by the known percentage of NK cells for each patient.

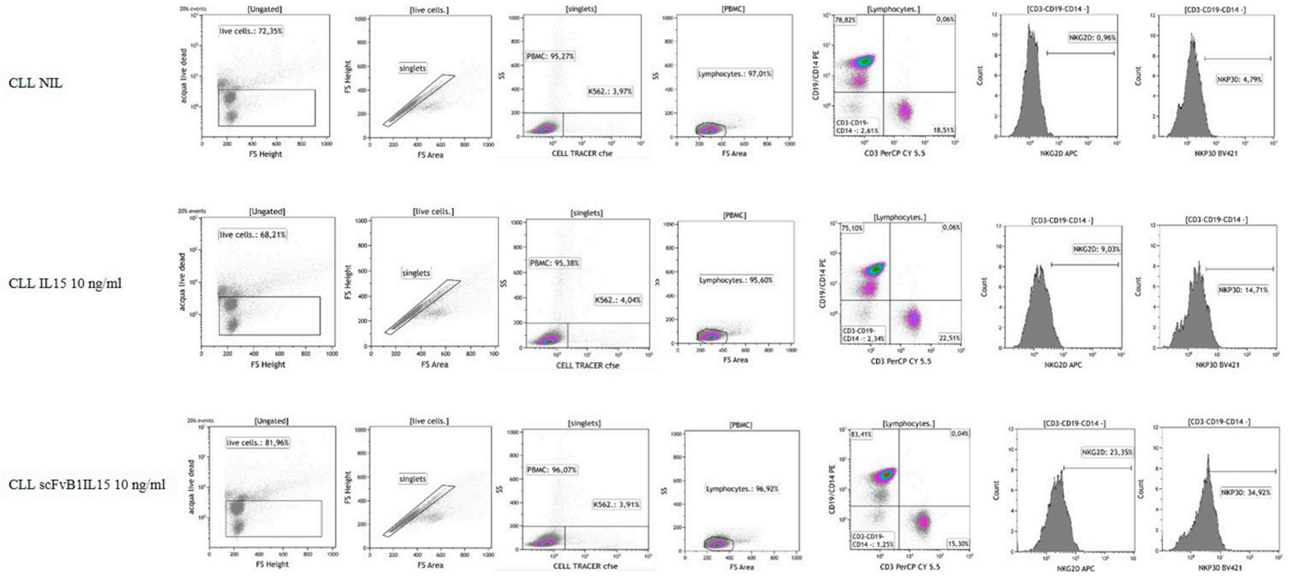

Supplementary Figure S10. Gating strategy for PBMCs from CLL patients (receptors). Gating strategy for expression of surface activating receptors (NKG2D and NKp30) in the NK cells; the figure shows the NK cell analysis in non-treated (NIL), IL15-treated, and scFvB1IL15-treated PBMCs from a representative CLL patient.

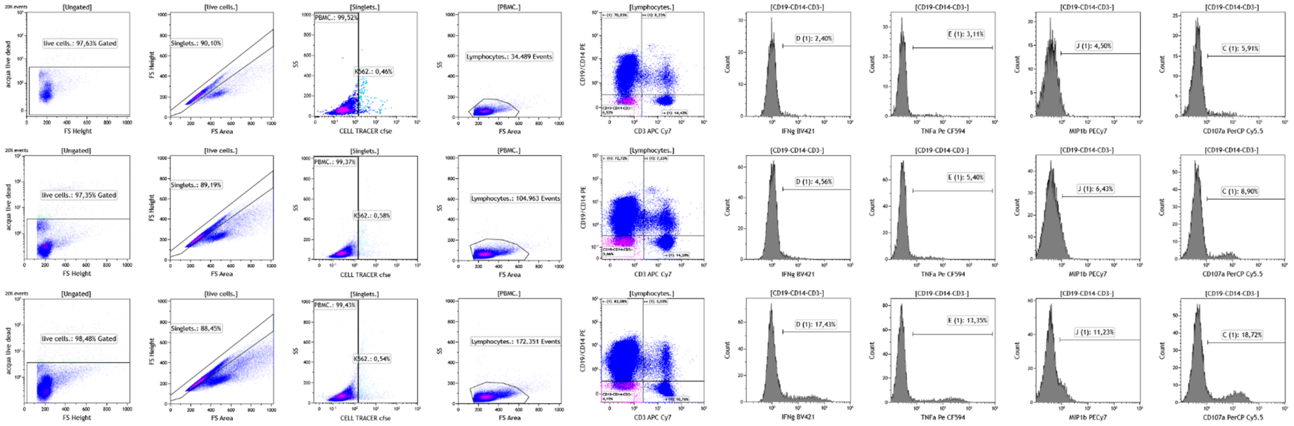

Supplementary Figure S11. Gating strategy for PBMCs from CLL patients (intracellular markers). Gating strategy for a representative CLL patient for the comparison of intracellular cytokines and CD107a marker in untreated, IL15-treated, and scFvB1IL15-treated PBMCs.
